# Loss of ADAMTS4 reduces high fat diet-induced atherosclerosis and enhances plaque stability in ApoE^−/−^ mice

**DOI:** 10.1038/srep31130

**Published:** 2016-08-05

**Authors:** Saran Kumar, Mo Chen, Yan Li, Fiona H. S. Wong, Chung Wee Thiam, Md Zakir Hossain, Kian Keong Poh, Satoshi Hirohata, Hiroko Ogawa, Véronique Angeli, Ruowen Ge

**Affiliations:** 1 Department of Biological Sciences, National University of Singapore, Singapore, 117543, Singapore; 2 Department of Microbiology, Yong Loo Lin School of Medicine, National University of Singapore, Singapore, 117456, Singapore; 3 Cancer Science Institute of Singapore, Singapore, 117599, Singapore; 4 Department of Medicine, Yong Loo Lin School of Medicine, National University of Singapore, Singapore; 5 Department of Cardiology, National University Heart Centre, National University Health System, Singapore 119228, Singapore; 6 Department of Medical Technology, Graduate School of Health Sciences, Okayama University, Okayama 700-8558, Japan; 7 Department of General Medicine, Graduate School of Medicine, Dentistry, and Pharmaceutical sciences, Okayama University, Okayama 700-8558, Japan

## Abstract

Atherosclerosis is a chronic inflammatory disease characterized by formation of lipid-rich plaques on the inner walls of arteries. ADAMTS4 (a disintegrin-like and metalloproteinase with thrombospondin motifs-4) is a secreted proteinase that regulates versican turnover in the arterial wall and atherosclerotic plaques. Recent reports indicated elevated ADAMTS4 level in human atherosclerotic plaques and in the plasma of acute coronary syndrome patients. Nevertheless, whether increased ADAMTS4 is a consequence of atherosclerosis or ADAMTS4 has a causal role in atherogenesis remains unknown. In this work, we investigated the role of ADAMTS4 in diet induced atherosclerosis using apolipoprotein E deficient (ApoE^−/−^) and *Adamts4* knockout mice. We show that ADAMTS4 expression increases in plaques as atherosclerosis progresses in ApoE^−/−^ mice. ApoE^−/−^Adamts4^−/−^ double knockout mice presented a significant reduction in plaque burden at 18 weeks of age. Loss of ADAMTS4 lead to a more stable plaque phenotype with a significantly reduced plaque vulnerability index characterized by reduced lipid content and macrophages accompanied with a significant increase in smooth muscle cells, collagen deposition and fibrotic cap thickness. The reduced atherosclerosis is accompanied by an altered plasma inflammatory cytokine profile. These results demonstrate for the first time that ADAMTS4 contributes to diet induced atherosclerosis in ApoE^−/−^ mice.

Atherosclerosis, the hardening and narrowing of the arteries, is the major cause for stroke and myocardial infarction^1^,^2^. Atherosclerosis is an inflammatory disorder induced by dyslipidemia^3^. It is a sequential, chronic, complex, multifactorial disease. Atherosclerotic plaques are composed of cells (macrophages, smooth muscle cells, and endothelial cells), lipids, extracellular matrix (ECM) and debris^4^,^5^. In advanced stages, the plaque may break off or rupture resulting in aggregation of platelets and the formation of thrombus. Thus atherosclerotic plaque can be classified into two types– stable or unstable (vulnerable). Characteristics of a vulnerable plaque include – 1) thin fibrous cap, 2) large necrotic core, 3) elevated inflammation, 4) increased vascularization, and 5) tissue proteolysis, 6) paucity of collagen and SMCs. Unstable plaques are the major causes of coronary artery diseases (CADs) as the thrombus dislocated from the plaque goes into circulation and blocks artery at narrow vessels, resulting in stroke or ischemia^6^,^7^.

ADAMTS4 (A disintegrin-like and metalloproteinase with thrombospondin motifs-4) is a secreted metalloproteinase of the ADAMTS family. It cleaves various ECM proteoglycans including aggrecan, brevican and versican^8^. It has been extensively studied for its role in the degradation of aggrecan in joint cartilage of osteoarthritis^9^. Recently, its role in angiogenesis and cancer has also been demonstrated^10^. Versican is a prominent component of arterial wall and is known to have critical importance in the formation of atherosclerotic lesions, with roles in lipid accumulation, inflammation and thrombosis^11^,^12^. On the other hand, versican degradation maybe linked to atherosclerotic lesion regression^12^.

A possible link between ADAMTS4 and atherosclerosis first emerged in 2008 when ADAMTS4 was shown to be elevated during the progression of atherosclerosis in LDLR^−/−^ApoB^100/100^ mice and in macrophage rich areas of human atherosclerotic plaques^13^. Subsequently, macrophages and smooth muscle cells (SMC) were reported to express ADAMTS4 in human atherosclerotic lesions^14^. ADAMTS4 is also elevated in human plasma of patients with both stable coronary artery diseases (CAD) as well as acute coronary syndromes (ACS)^15^. High plasma levels of ADAMTS4 is also associated with the severity of CADs in patients^16^,^17^. More importantly, using a tandem stenosis induced carotid artery plaque rupture model in ApoE^−/−^ mice, potential pathogenic factors that are upregulated in ruptured plaques were identified. ADAMTS4 turned out to be one of the top hits with a 7.9-fold increase in unstable plaques^18^. Nevertheless, none of these studies revealed whether elevation in ADAMTS4 level is a consequence of atherosclerosis or ADAMTS4 has a causal role in atherogenesis.

In this work, we experimentally clarified if ADAMTS4 plays a role in atherosclerosis. Using high fat diet induced atherosclerosis in ApoE^−/−^ mouse, we generated *Adamts4* and *ApoE* double knockout mice (ApoE^−/−^Adamts4^−/−^). As CADs present many gender-related differences, we investigated atherogenesis in both male and female mice^19^. Our results demonstrate that loss of ADAMTS4 attenuated diet induced atherosclerosis with significantly reduced plaque burden in ApoE-deficient mice. Furthermore, plaques developed in the ApoE^−/−^Adamts4^−/−^ environment exhibited reduced lipid content, decreased macrophages, declined versican degradation, with concomitant increase in SMCs and collagen deposition, all together presenting characteristics of more stable plaques.

## Results

### Loss of *Adamts4* does not affect plasma lipid profile in ApoE^−/−^ mice

ApoE^−/−^ mice develops hypercholesterolemia and complex atherosclerotic plaques that closely mimic human lesions^20^,^21^. To clarify the role of ADAMTS4 in atherosclerosis, we generated double knockout mice (ApoE^−/−^Adamts4^−/−^) by crossing Adamts4^−/−^ mice with ApoE^−/−^ mice (both in C57Bl/6J background). ApoE^−/−^Adamts4^−/−^ genotype was confirmed by genotyping with genomic PCR. Mice were fed on Western-type high fat diet starting from 6 weeks of age and were sacrificed at two time points – 12 and 18 weeks of age. No significant difference between the body weight of the ApoE^−/−^Adamts4^−/−^ mice compared to that of ApoE^−/−^ mice. As expected, feeding on high fat diet lead to a 7–8 fold increase of plasma cholesterol in ApoE^−/−^ and ApoE^−/−^Adamts4^−/−^ mice compared to control C57BL/6J mice. However, no significant difference in plasma cholesterol was observed between ApoE^−/−^ and ApoE^−/−^Adamts4^−/−^ mice of both 12 and 18 weeks of age. Adamts4 deletion also did not influence triglycerides or HDL in the sex and age matched groups (Supplementary Table S1).

### Genetic ablation of *Adamts4* reduces diet-induced atherosclerosis in ApoE^−/−^ mice

We next analysed the effect of *Adamts4* knockout on high fat diet induced atherosclerosis in ApoE^−/−^ mice. ApoE^−/−^Adamts4^−/−^ mice fed on Western diet were sacrificed at 12 or 18 weeks of age and compared with the age and sex matched ApoE^−/−^ or C57BL/6J wild-type control. Direct observation of the aortic arch, brachiocephalic artery and the carotid artery under a stereomicroscope showed no morphometry differences at 6 weeks of age in all mouse groups before the commencement of high-fat diet (Supplementary Fig. S1). This rules out the possibility that *Adamts4* knockout directly lead to perturbations in vessel morphology which could affect atherogenesis in ApoE^−\−^ mice.

**Figure 1:**
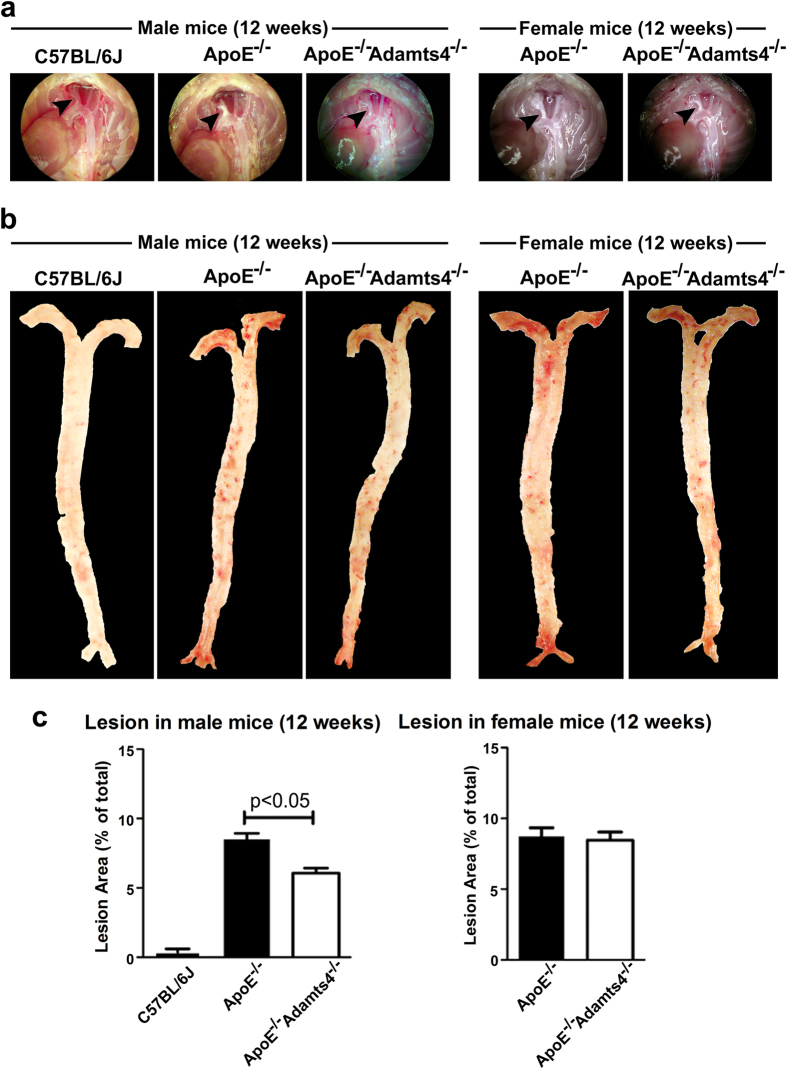
Comparison of atherosclerosis in 12-weeks old ApoE^−/−^ and ApoE^−/−^Adams4^−/−^ mice. (**a**) Photos of the aortic arch in wildtype, ApoE^−/−^ and ApoE^−/−^Adams4^−/−^ mice. Arrowhead indicates the brachiocephalic artery region with prominent lesion. (**b**) *en face* ORO stained main aortic tree (from the ascending aorta to the iliac bifurcation devoid of any branches) of 12-weeks old mice. (**c**) Quantification of the ORO positive area represented as percentage of total arterial trunk area (n = 8 for all ApoE^−/−^ and ApoE^−/−^Adams4^−/−^ mice, n = 4 for C57BL/6J male control mice). Values shown are mean ± SEM.

**Figure 2:**
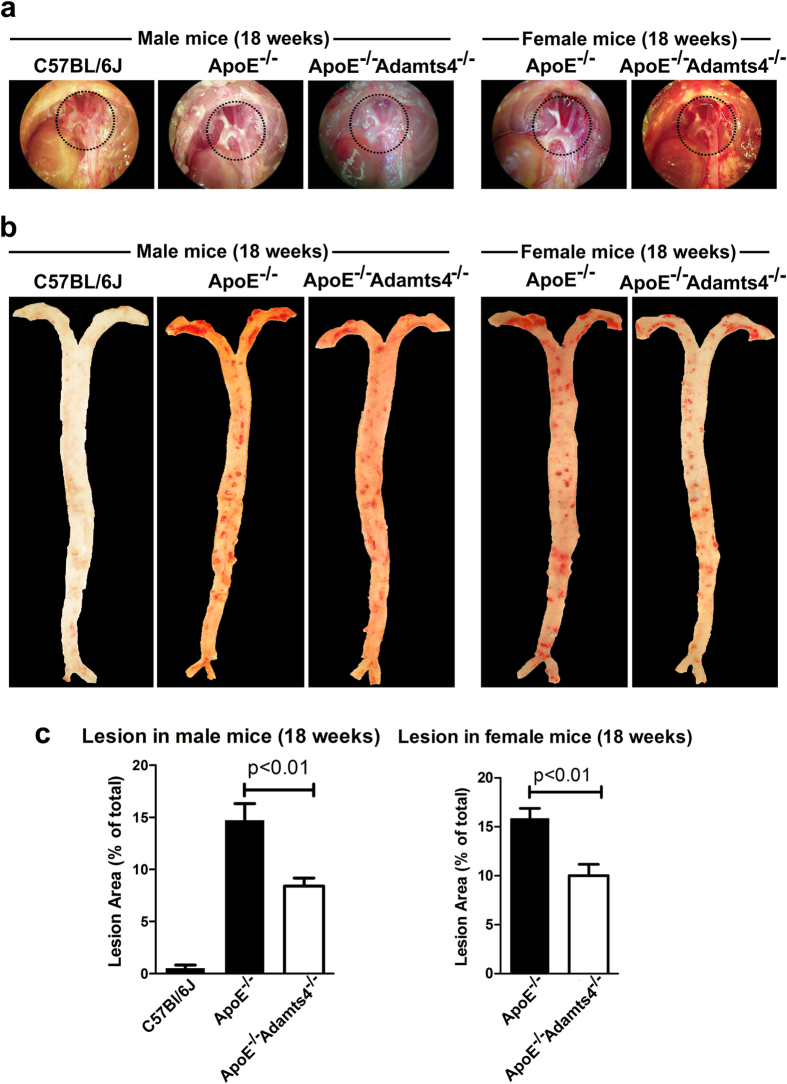
Reduced atherosclerotic lesions in 18-weeks old ApoE^−/−^Adamts4^−/−^ mice. (**a**) Photos of the aortic arch. Dotted circle indicates the aortic arch region with prominent lesion. (**b**) *en face* ORO stained main trunk of the aortic tree of 18-week old mice. (**c**) Quantification of the ORO positive area of the *en face* images shown in panel b (n = 8 for all ApoE^−/−^ and ApoE^−/−^Adams4^−/−^ mice, n = 4 for C57BL/6J male control mice).

No sites of predilection for lesion formation in wild-type C57BL/6J male group were observed in both 12 and 18 weeks age group (Figs 1a and 2a). However, in ApoE^−/−^ mice, atherosclerosis was most prominent in aortic root, brachiocephalic artery followed by the aortic arch and carotid artery branches. As expected, there was a progression of lesion development with age i.e., from 12 to 18 weeks under high fat diet (Figs 1a and 2a). Compared with 12-weeks old ApoE^−/−^ mice, reduced atherosclerosis was observed in ApoE^−/−^Adamts4^−/−^ male mice but not female mice (Fig. 1a). By 18-weeks of age, absence of ADAMTS4 resulted in obvious decrease in plaque formation in both male and female mice (Fig. 2a).

*En face* analysis of Oil Red O (ORO) stained main arterial trunk (from the ascending aorta to the iliac bifurcation without including any branches) revealed that no lesions in C57BL/6J wild-type male mice of both 12- and 18-weeks age group. In contrast, atherosclerotic lesions were observed in ApoE^−/−^ mice and they were most prominent within the ascending aorta, the aortic arch and the descending aorta (Figs 1b and 2b). The atherosclerotic lesions in 12-weeks old male ApoE^−/−^Adamts4^−/−^ mice was significantly reduced compared to their corresponding ApoE^−/−^ male mice. However, this difference was not obvious in 12 weeks-old female group (Fig. 1b,c; Table 1). In 18-weeks old mice, significant reduction of plaques were observed in both male and female ApoE^−/−^Adamts4^−/−^ mice compared to that of sex and age matched ApoE^−/−^ mice (Fig. 2b,c; Table 1). Hence, removal of Adamts4 attenuates diet induced atherosclerosis in ApoE^−/−^ mice.

### Loss of ADAMTS4 in ApoE^−/−^ mice lead to more stable plaques

**Figure 3:**
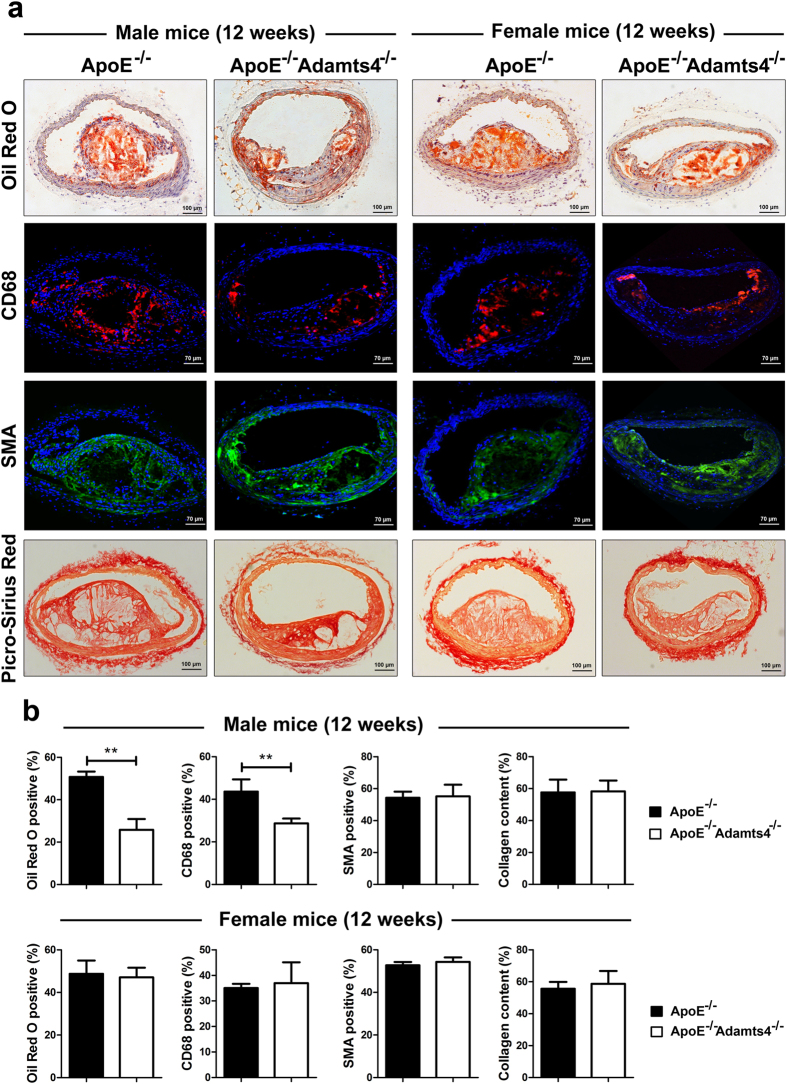
Lesion morphology of the brachiocephalic artery in 12-weeks old mice. (**a**) Representative images of 7 μm sections of brachiocephalic artery stained for ORO, CD68, SMA and collagen. (**b**) Quantification of the staining represented as a percentage of stained area over the complete plaque area (n = 5 mice, with 5 sections from each brachiocephalic trunk). Values shown are mean ± SEM.

**Figure 4:**
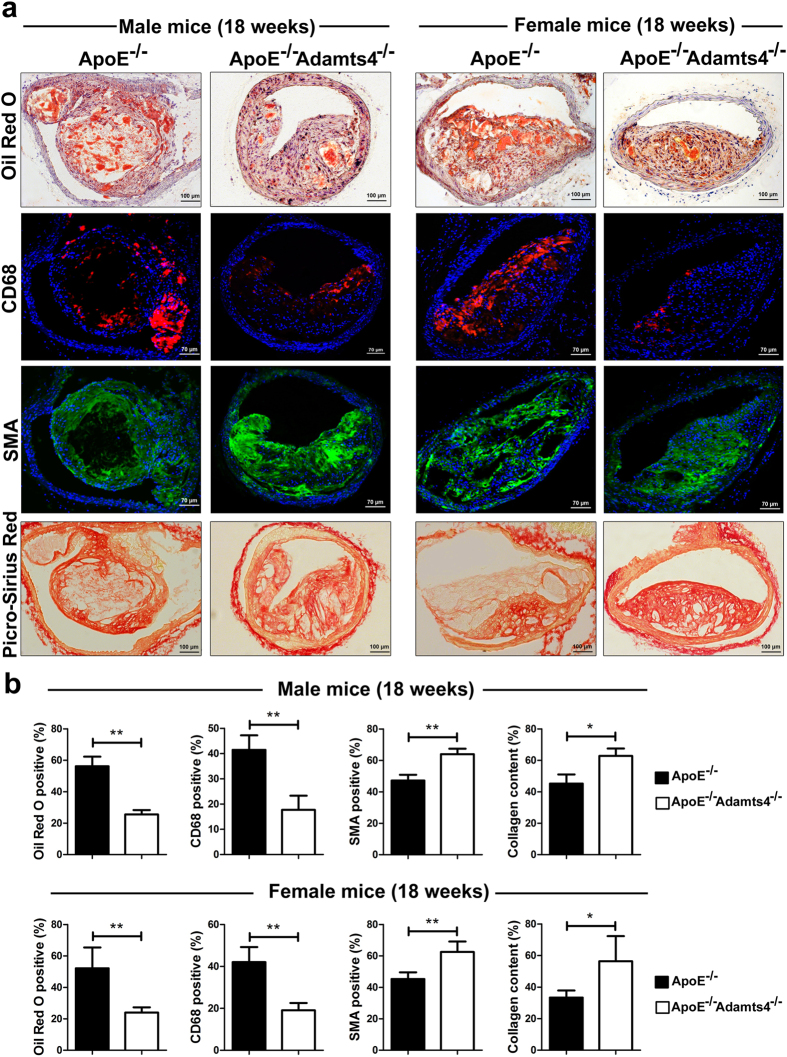
Brachiocephalic artery plaque assessment in 18-weeks old mice. (**a**) Representative images of brachiocephalic artery stained for ORO, CD68, SMA and collagen. (**b**) Quantification of the staining represented as a percentage of stained area over the complete plaque area (n = 5 mice, with 5 sections from each brachiocephalic trunk). Values shown are mean ± SEM.

Plaques from the brachiocephalic artery were analysed for plaque composition by staining the cross sections. Lipid content in the plaques was determined by ORO staining of the cross sections. About 50% decrease in lipid deposition was observed in 12-weeks old male ApoE^−/−^Adamts4^−/−^ mice compared to that of ApoE^−/−^ mice (Fig. 3). However, at this age, no significant difference was observed in the female counterpart (Fig. 3). Similarly, lipid content was reduced to half in 18-weeks male ApoE^−/−^Adamts4^−/−^ mice compared to that in ApoE^−/−^ mice. Notably, female ApoE^−/−^Adamts4^−/−^ mice in the 18-weeks group also presented with a 54% reduction in lipid deposition compared to ApoE^−/−^ mice (Fig. 4).

By immunostaining with macrophage marker CD68, significant reduction of macrophages within plaques were observed in 12-weeks old male ApoE^−/−^Adamts4^−/−^ mice compared to that of ApoE^−/−^ mice(Figs 3 and 4, Table 1). In contrast, no significant difference was observed in female mice of the same age (Fig. 3a,b). In 18-weeks old mice, both male (by 57%) and female (by 55%) showed a significant reduction in plaque macrophage content in the absence of Adamts4 (Fig. 4a,b, Table 1).

No significant difference in the SMC content of the plaques was observed in 12-weeks old age group (Fig. 3). However, in 18-weeks old mice, a significant increase (36% and 38% respectively) in SMC content was observed in plaques from ApoE^−/−^Adamts4^−/−^ mice of both sexes (Fig. 4). SMA staining of the media of the aortic wall is clearly visible when the image is focused on the media, indicating the reliability of SMA staining in the plaques (Fig. S2). Collagen contents in the plaques were determined by Picro-Sirius red staining of the tissue sections. While similar level of collagen were observed in plaques between mice in 12-weeks old groups, a marked increase in collagen content (39% and 69% respectively) was observed in 18-weeks old ApoE^−/−^Adamts4^−/−^ mice in both male and female groups (Fig. 4).

In humans, plaque rupture is the major cause of acute myocardial infarction and stroke^7^. Spontaneous and reproducible plaque rupture has been reported in ApoE^−/−^ mice under long-term fat-feeding. These plaque ruptures were reported to occur predominantly in the brachiocephalic artery branches^22^. The proteinase activity of ADAMTS4 has been postulated to promote plaque instability/vulnerability^18^. To investigate the possible effect of ADAMTS4 removal on plaque stability, we determined the thickness of the fibrous cap, lipid rich necrotic core, the ratio of fibrous cap to necrotic core (cap/core ratio) and the plaque vulnerability index in various mouse groups^6^,^7^,^23^,^24^. In the brachiocephalic arterial plaques of 12-weeks old mice, no significant difference in cap thickness or cap/core ratio was observed between ApoE^−/−^ and ApoE^−/−^Adamts4^−/−^ mice (Table 1). However, there was a significant decrease (>50%) in the necrotic core in male mice, but not in female mice (Supplementary Fig. S3). Similarly, plaque vulnerability index was also significantly reduced in 12-weeks old male mice, but not in female mice (Table 1). In 18-weeks old mice groups, a significant increase (*P* < *0.05*) in the thickness of fibrous cap in plaques of ApoE^−/−^Adamts4^−/−^ mice was observed relative to sex matched ApoE^−/−^ mice (Table 1). Meanwhile, a significant decrease (around 50%) in the necrotic area (% of total plaque area) was observed in both male and female mice (Supplementary Fig. S3). In 18-weeks old mice, we also observed a significant increase (*P* < *0.05*) in cap/core ratio of both male (0.09 ± 0.01) and female ApoE^−/−^Adamts4^−/−^ mice (0.08 ± 0.01) compared to their age, sex matched ApoE^−/−^ mice (male: 0.05 ± 0.01, female: 0.04 ± 0.01). Moreover, ApoE^−/−^ male mice had a 2-fold higher plaque vulnerability index (1.06 ± 0.13) compared to that of ApoE^−/−^Adamts4^−/−^ mice (0.34 ± 0.05) (*P* < *0.01)*. In 18-weeks old female group, absence of ADAMTS4 reduced the plaque vulnerability index to one-third (ApoE^−/−^: 1.22 ± 0.31 to ApoE^−/−^Adamts4^−/−^: 0.37 ± 0.07) (*P* < 0.01) (Table 1).

Altogether, the above plaque characteristics in ApoE^−/−^Adamts4^−/−^ mice depict a more stable plaque phenotype.

### ADAMTS4 expression is up-regulated in plaques and plasma of ApoE^−/−^ mice as atherosclerosis progresses

**Figure 5:**
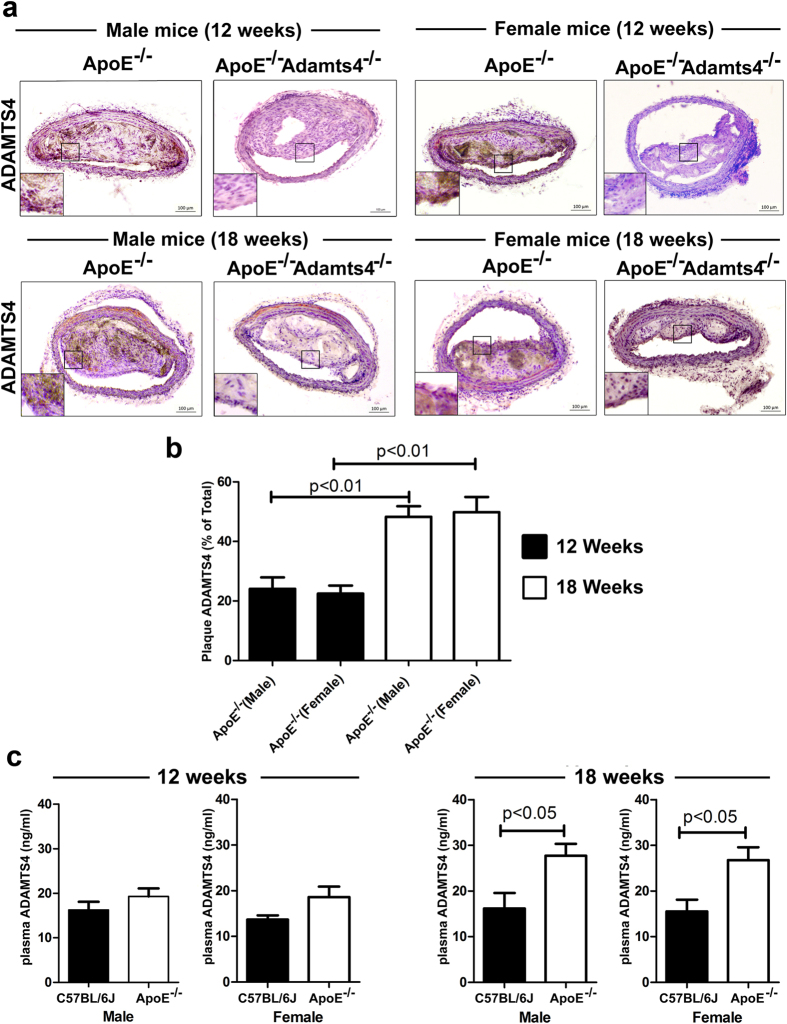
Elevated levels of ADAMTS4 in plaques and plasma of ApoE^−/−^ mice as atherosclerosis progresses. (**a**) ADAMTS4 expression in the plaques of 12 and 18 weeks old ApoE^−/−^ mice. (**b**) Quantification of the ADAMTS4 positive stained area expressed as percentage of total lesion area (n = 5 mice, with 5 sections from each brachiocephalic artery). (**c**) Plasma ADAMTS4 level in ApoE-null mice (n = 8). Values shown are mean ± SEM.

The above results clearly demonstrate that the absence of ADAMTS4 caused a much more significant reduction in atherosclerosis in the 18-weeks old mice than that of the 12-weeks age group. It has been previously reported that ADAMTS4 protein level is elevated in the plasma of CAD patients^15^,^18^ and in the plaques of human carotid atherosclerotic lesions^13^,^18^. In addition, ADAMTS4 is elevated in plaques of LDLR^−/−^ApoB^100/100^ mice^13^. To investigate if ADAMTS4 accumulation in plaques correlates with the progression of atherosclerosis in ApoE^−/−^ mice, we analysed ADAMTS4 expression in plaques by immunohistochemistry (IHC). As shown in Fig. 5, ADAMTS4 is present in the plaques of ApoE^−/−^ mice of both age groups (Fig. 5a,b). There was a 2-fold increase in ADAMTS4 expression in the plaques of the 18 weeks old ApoE^−/−^ mice compared to that of the 12 weeks old ApoE^−/−^ mice of the same sex (Fig. 5a,b). Furthermore, ADAMTS4 was detected in the plasma of both 12- and 18-weeks old ApoE^−/−^ mice, with significantly higher levels in 18-weeks old mice compared to that of the wildtype mice (Fig. 5c). Hence, an obvious increase in plasma ADAMTS4 protein was also observed as atherosclerosis progresses in ApoE^−/−^ mice from 12-weeks old to 18-weeks old. Accordingly, ApoE^−/−^Adamts4^−/−^ mice showed a loss of ADAMTS4 expression in plaques (Fig. 5a).

### Macrophages and SMCs in atherosclerotic plaques express ADAMTS4

**Figure 6:**
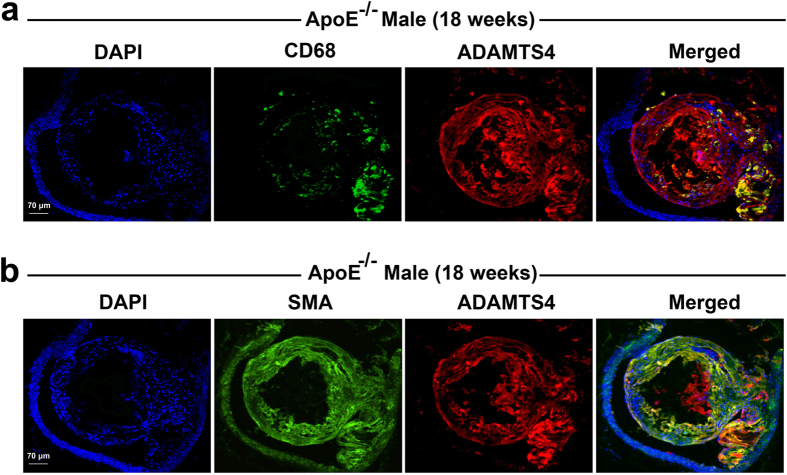
Cells expressing ADAMTS4 in the plaque. Co-localization of ADAMTS4 with CD68 (**a**) and SMA (**b**) in brachiocephalic aortic plaques of 18 weeks old ApoE^−/−^ mice.

During atherogenesis, monocytes from the circulating blood infiltrate into the intima of the vessel wall and differentiate into macrophages, a major component of the atherosclerotic plaques^25^. This process is very much dependent on proteases that facilitate cell migration and invasion^26^. Since ADAMTS4 is abundantly expressed in the atherosclerotic plaques of ApoE^−/−^ mice, we assessed what cells in plaques express ADAMTS4 by immunofluorescent (IF) staining. As shown in Fig. 6, ADAMTS4 co-localized with CD68 positive region in the plaques of 18-weeks old ApoE^−/−^ mice, suggesting that macrophages express ADAMTS4 (Fig. 6a). In addition, ADAMTS4 co-localized with smooth muscle actin (SMA) positive region of plaques, suggesting that SMCs in lesions also express ADAMTS4 (Fig. 6b). These findings are consistent with previous reports in LDLR^−/−^ApoB^100/100^ mouse model as well as in human atherosclerotic lesions.

### Loss of ADAMTS4 influences the proliferation/apoptosis of intra-plaque macrophages and smooth muscle cells

Once it was established that ADAMTS4 is expressed in macrophages and SMCs of atherosclerotic plaques, we further examined the proliferation/apoptosis of intra-plaque macrophages and SMCs upon loss of ADAMTS4 by immunofluorescent staining. Plaque cross-sections from all four 18-weeks old mice groups were triple stained for CD68, SMA and Ki67 (marker of cell proliferation). Quantification of CD68/SMA and Ki67 double positive area against CD68/SMA positive area shows that removal of ADAMTS4 significantly reduced macrophage proliferation but enhanced smooth muscle cells proliferation (Supplementary Fig. S4). Apoptosis of macrophages and smooth muscle cells was determined by triple staining of CD68, SMA and terminal deoxynucleotidyl transferase dUTP nick end labelling (TUNEL, the marker for cell apoptosis). The quantification of CD68/SMA and TUNEL double positive area against CD68/SMA positive area shows that ADAMTS4 deletion significantly induced macrophage apoptosis but has no effect on the apoptosis of smooth muscle cells (Supplementary Fig. S5). These findings indicate that upon knockout of *Adamts4*, the reduced intra-plaque macrophages are due to a combined effect of reduced proliferation and increased apoptosis while the increased intra-plaque smooth muscle cells are due to enhanced proliferation only.

### ADAMTS4 deficiency alters inflammatory cytokine profile in the blood of ApoE^−/−^ mice

**Figure 7:**
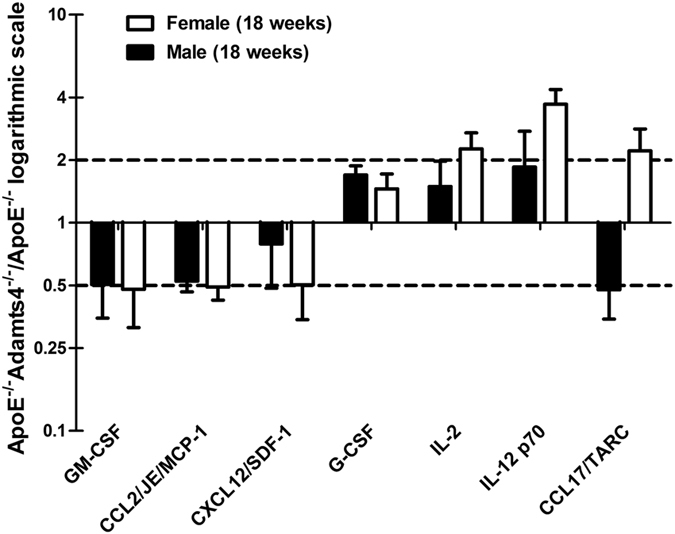
Plasma cytokine profile change in ApoE^−/−^Adams4^−/−^ mice. Comparison of cytokines in the plasma from 18 weeks old mice (n = 3, each sample is a pool of blood plasma from 4 mice). Values shown are mean ± SEM.

As inflammation is a major contributing factor in atherosclerosis progression and plaque instability, we next analysed if absence of ADAMTS4 influences the plasma inflammatory cytokines between 18 weeks old sex matched ApoE^−/−^ and ApoE^−/−^Adamts4^−/−^ mice. The relative expression of 40 cytokines in the plasma was determined. A total of 7 cytokines were found to be significantly deregulated when ADAMTS4 is lost (Fig. 7). Three pro-inflammatory cytokines namely granulocyte-macrophage colony-stimulating factor (GM-CSF), C-C motif chemokine ligand 2 (CCL2) (also referred to as macrophage chemoattractant protein-1 [MCP-1]), and C-X-C motif chemokine 12 (CXCL12) (also called stromal cell-derived factor 1[SDF-1]), were significantly down-regulated about 50% in the plasma when ADAMTS4 is absent (Fig. 7). On the other hand, three other cytokines namely granulocyte colony-stimulating factor (G-CSF), interleukin-2 (IL-2) and interleukin-12 were up-regulated more than 1.5 fold in ApoE^−/−^Adamts4^−/−^ mice (Fig. 7). Notably, both IL-2 and IL-12 have been reported to be pro-atherogenic cytokines^27^,^28^,^29^,^30^. Interestingly, we also observed one C-C motif chemokine ligand 17 (CCL17) also known as thymus and activation regulated chemokine (TARC) which is differentially regulated between male and female mice (Fig. 7). Hence, ADAMTS4 seems to promote atherosclerosis at least partially by promoting the production of pro-inflammatory cytokines such as GM-CSF, CCL2, and CXCL12, all of which function in promoting monocyte/macrophage recruitment, differentiation, proliferation or survival^25^,^31^,^32^,^33^,^34^,^35^.

### Knockout of *Adamts4* leads to decreased versican and aggrecan cleavage in atherosclerotic plaques of ApoE^−/−^ mice

**Figure 8:**
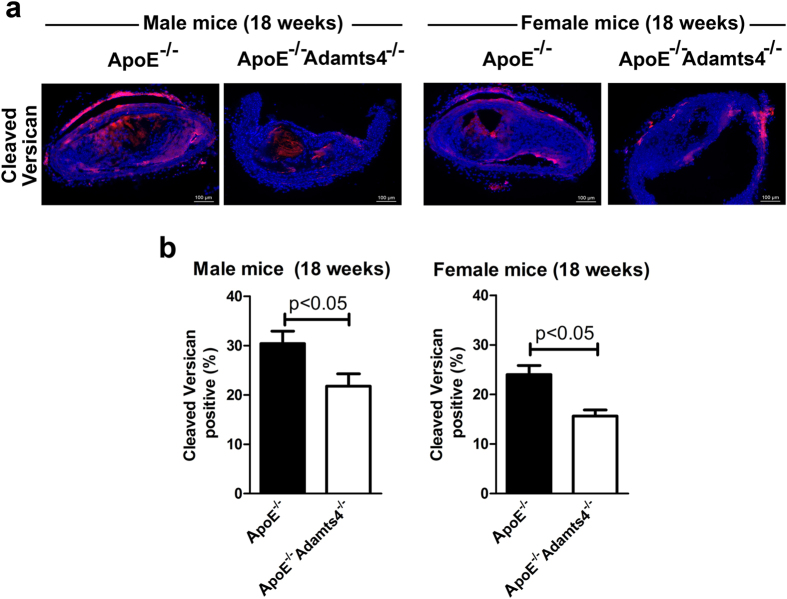
Versican degradation in ApoE^−/−^Adams4^−/−^ mice. (**a**) Representative image of the brachiocephalic artery section stained with versican neoepitope antibody. (**b**) Quantification of degraded versican (n = 5, 5 sections per brachiocephalic trunk).

As mentioned above, versican is a major proteoglycan of the ECM of atherosclerotic lesions and is known to play critical roles in plaque lipid accumulation, inflammation and thrombosis^12^. ADAMTS4 is known to cleave versican V1 at its N-terminal G1 domain generating a neo-epitope DPEAA^11^,^36^,^37^. Hence, we examined versican degradation in atherosclerotic plaques of ApoE^−/−^ and ApoE^−/−^Adamts4^−/−^ mice using antibody recognizing this neoepitope. As shown in Fig. 8, versican degradation was significantly lower in plaques of ApoE^−/−^Adamts4^−/−^ mice compared to that of ApoE^−/−^ mice. Thus, the reduction of versican cleavage in plaques of ApoE^−/−^Adamts4^−/−^ mice correlated with decreased plaque burden and lipid content.

Aggrecan is the other major proteoglycan substrate for ADAMTS4^38^. ADAMTS4 cleaves aggrecan in its interglobular domain (Glu373-Ala374), resulting in the release of aggrecan fragments^39^. We studied the aggrecan degradation in plaques of ApoE^−/−^ and Adamts4^−/−^ApoE^−/−^ mice using an antibody that recognizes the N-terminal neoepitope ARG. As expected, a significant decrease in aggrecan cleavage within the plaques was observed in Adamts4^−/−^ApoE^−/−^ mice at 18 weeks of age in both sexes (Supplementary Fig. S6).

### No compensatory upregulation of ADAMTS1, -5 and -8 upon loss of ADAMTS4 in ApoE^−/−^ mice

We examined and compared the protein expression of three ADAMTS family members that are closely related to ADAMTS4, namely ADAMTS1, -5 and -8. Loss of ADAMTS4 did not change the plasma levels of these ADAMTSs between ApoE^−/−^ and ApoE^−/−^Adamts4^−/−^ mice at 6 weeks of age before the start of the high fat diet (Supplementary Fig. S7). In addition, IF staining of the cross sections of brachiocephalic artery plaques revealed no alterations of protein levels of these three ADAMTSs in the absence of ADAMTS4 (Supplementary Fig. S8). Hence, it is unlikely that any significant compensatory changes occurred of these three ADAMTSs in either plaques or blood circulation upon loss of ADAMTS4. It is noted that the expression of ADAMTS1, -5 and -8 are much lower in the plaques comparing to ADAMTS4.

## Discussion

The salient finding of the present study is that elimination of ADAMTS4 attenuates atherosclerotic plaque formation and reduces plaque vulnerability in high fat diet induced atherosclerosis of ApoE^−/−^ mice. This finding experimentally delineates that ADAMTS4 is a contributor to atherosclerosis and a potential biomarker for unstable plaques. Our findings are consistent with the previous report that ADAMTS4 is highly upregulated in unstable plaques in a tandem stenosis induced plaque rupture model in ApoE^−/−^ mouse^14^.

Although no changes in the plasma lipids between ApoE^−/−^ and ApoE^−/−^Adamts4^−/−^ mice were observed (Supplementary Table S1), removal of ADAMTS4 attenuates atherosclerosis (Figs 1 and 2). This effect is not due to any direct impact on vessel morphometry by *Adamts4* ablation since no alteration of the vessel morphology were observed between ApoE^−\−^Adamts4^−\−^ and ApoE^−\−^ mice before the commencement of high-fat diet at 6 weeks of age (Supplementary Fig. S1). In addition, a significant reduction in plaque vulnerability index was observed when ADAMTS4 is removed (Table 1), indicating a role for ADAMTS4 in promoting plaque instability.

We found that ADAMTS4 is expressed in plaques and this expression increases as atherosclerosis progresses. Both macrophages and SMCs within the plaques express ADAMTS4 (Fig. 6). Our finding is consistent with previously reported ADAMTS4 up-regulation during monocyte to macrophage differentiation^13^ and the expression of this gene in macrophages during thoracic aortic aneurysms^14^,^37^. In addition, elevated levels of ADAMTS4 were also observed in peripheral monocytes in human acute coronary syndrome patients^15^. In the absence of ADAMTS4, we observed an obvious reduction of macrophages within the atherosclerotic lesions (Figs 3 and 4). It was previously reported that TGF-β up-regulates ADAMTS4 expression in cultured macrophages and increases macrophage infiltration into ECM. On the other hand, knockdown of ADAMTS4 reduced macrophage invasion into ECM *in vitro*^14^. The reduction of macrophages within the plaques of ApoE^−/−^Adamts4^−/−^ mice suggested a possible reduction of monocyte (macrophage) infiltration into the plaques since all macrophages in atherosclerotic plaques come from blood monocytes^40^.

Vascular smooth muscle cells (SMCs) also play a critical role in atherosclerosis and are the main source of ECM including versican within atherosclerotic lesions^12^,^41^. Although proliferation and migration of SMCs facilitates early lesion development, they are also critical in maintaining plaque stability by maintaining a protective fibrous cap in advanced lesions. Several factors including growth factors, inflammatory cytokines and matrix metalloproteinases (MMPs) have been shown to influence SMC growth during atherosclerosis^42^. We showed here that ADAMTS4 is expressed in plaque SMCs of ApoE^−/−^ mice (Fig. 6). In ApoE^−/−^Adamts4^−/−^ mice, a significant reduction of cleaved versican in the atherosclerotic plaques was observed (Fig. 8). Versican cleavage has been linked to increased SMC death in atherosclerotic plaques^36^. ADAMTS4 mRNA has been shown to significantly increase under the condition of enhanced SMC death and versican cleavage^36^. SMC death results in the loss of the fibrous cap, leading to plaque instability^43^. Consistently, removal of ADAMTS4 lead to an increase in SMCs and a decrease in versican cleavage, a phenotype reflective of more stable plaques (Fig. 4). Thus, it is highly likely that both macrophages and SMCs are culpable cells for atherogenesis.

To further investigate the effect of ADAMTS4 on macrophages and SMCs in plaques, we compared the proliferation and apoptosis of these two cell types in the plaques of 18 week old ApoE and ApoEAdamts4 null mice by co-immunostaining. The impact of ADAMTS4 loss was more pronounced for plaque macrophages where we observed a significant reduction in proliferation coupled with increased apoptosis. In case of SMCs, ADAMTS4 deletion significantly induced proliferation but had little effect on SMC apoptosis (Supplementary Figs S4 and S5). Taken together, the role of ADAMTS4 in atherogenesis is primarily through macrophages followed by SMCs in ApoE null background. However, further studies are warranted to decode the mechanism which leads to perturbation of cell death and proliferation. Downstream targets of this metalloproteinase such as the proteoglycans (versican, aggrecan) or other receptor dependent cell signalling would be worth investigating to understand how ADAMTS4 promotes atherogenesis.

Furthermore, collagen deposition in late stage atherosclerosis is required for structural support of the fibrotic cap to avoid plaque rupture^44^. SMCs are the main source of collagen in plaques^45^. An increase of SMCs in ApoE^−/−^Adamts4^−/−^ mice correlates with a significant increase in total plaque collagen at 18-weeks old mice (Fig. 4). Altogether, removal of ADAMTS4 resulted in a net increase of SMC content and collagen deposition in plaques, characteristics of more stable plaques. Degradation of ECM components influences atherosclerosis at various stages by affecting cell migration and proliferation^46^. In addition to versican, aggrecan also is an ADAMTS4 substrate^47^ and it is an important ECM component present in plaques of ApoE^−/−^ mice^48^. Aggrecan degradation is also significantly inhibited in ApoE^−/−^Adamts4^−/−^ mice at 18 weeks in both sexes.

Thinning of the fibrotic cap of the plaque and an increase in the lesion’s necrotic area in conjunction with inflammation are known indicators of human vulnerable/unstable plaques^7^. Our results showed that removal of ADAMTS4 in ApoE deficient mice increased the thickness of the fibrous cap and cap/core ratio in 18-weeks old ApoE^−/−^Adamts4^−/−^ mice accompanied with significant reductions in necrotic core area (>30% in both sexes) (Supplementary Fig. S3). Consequently, plaque vulnerability index was dramatically reduced when ADAMTS4 is removed (Table 1).

The impact of ADAMTS4 removal on atherosclerosis is more striking in 18-weeks old mice compared to that of 12-weeks old mice (Figs 1, 2, 3, 4). Nevertheless, even in the 12 weeks old mice, a significantly reduced atherosclerosis was observed in ApoE^−/−^Adamts4^−/−^ male mice, albeit not in the female counterpart. Possible explanations are manifold. Firstly, gender specific differences are present in ApoE^−/−^ mice due to the differences in hormones, especially oestrogen, which might mask the effect of ADAMTS4^49^. Secondly, female mice have a more favourable lipid profile than male mice in total cholesterol, total triglycerides and increased HDL level (Supplementary Table S1). Thirdly, the pronounced effect of ADAMTS4 removal in 18-weeks old ApoE^−/−^ mice may be a result of higher ADAMTS4 expression in both the atherosclerotic lesions as well as the plasma comparing to that of 12-weeks old mice (Fig. 5). The increased ADAMTS4 expressions are consistent with a previous report in the LDLR^−/−^ApoB^100/100^ mice model of atherosclerosis^13^ as well as in patients with CAD^15^. Thus, the possibility of using plasma ADAMTS4 as a biomarker for atherosclerosis warrants further exploration.

Inflammation is a key regulator of atherosclerosis and plays critical role in the destabilization of the plaques^50^. Macrophages and SMCs not only secrete but also are activated by various cytokines^51^. We compared the blood profile of 40 known cytokines between ApoE^−/−^Adamts4^−/−^ mice and ApoE^−/−^ mice. Three known pro-atherogenic cytokines: GM-CSF, MCP-1 (CCL2) and SDF-1(CXCL12), are decreased for more than 50% when ADAMTS4 is removed (Fig. 7). GM-CSF, a cytokine facilitating monocytes to macrophages differentiation and a driver for plaque vulnerability^52^, is reported to be abundantly present in atherosclerotic lesions^32^,^53^ and promote advanced plaque progression in low-density lipoprotein-driven atherosclerosis in mice^54^. MCP-1, a pro-inflammatory cytokine produced by macrophages and SMCs, is known to enhance macrophage infiltration and is strongly associates with atherogenesis^33^. SDF-1 is a well-known chemotactic factor for monocytes and is expressed in atherosclerotic plaques^34^. Meantime, three other cytokines namely G-CSF, IL-2 and IL-12 are up-regulated >1.5 fold in ApoE^−/−^Adamts4^−/−^ mice. G-CSF treatment of ApoE^−/−^ mice has been reported to result in smaller atherosclerotic plaques, decreased ORO staining, and decreased infiltration of monocyte/macrophage into aortic lesions coupled with lower cholesterol levels^55^. Thus, upregulation of G-CSF may contribute to the reduced atherosclerotic lesion in ApoE^−/−^Adamts4^−/−^ mice. On the other hand, both IL-2 and IL-12 are known pro-atherogenic factors^27^,^28^,^29^,^30^. It is unclear how IL-2 and IL-12 upregulation influence atherosclerosis in ApoE^−/−^Adamts4^−/−^ mice and further studies are needed to clarify their roles.

CCL17 is a chemokine expressed by dendritic cells (DCs) that has been reported to promote atherosclerosis in a mechanism conferred by T cells^56^. CCL17 is present in advanced human and mouse atherosclerosis and CCL17 and DCs accumulate in atherosclerotic lesions. In ApoE^−/−^ mice, Ccl17 deficiency entailed a reduction of atherosclerosis, which was dependent on T-regulatory cells^56^. Intriguingly, we observed a differential regulation of CCL17 between male and female plasma in the absence of ADAMTS4. The significance of this observation is unclear at this stage. As we only investigated the cytokine profile of blood plasma but not that of the plaque itself, how ADAMTS4 influences cytokine changes in the plaque microenvironment need to be further studied in future. Nevertheless, the altered blood plasma cytokines may influence atherosclerosis and plaque vulnerability in ApoE^−/−^Adamts4^−/−^ mice.

Besides ADAMTS4, several other members of the ADAMTS proteinase family have also been implicated in atherogenesis. For example, ADAMTS1 has been reported to promote atherogenesis by cleaving versican and promoting SMC migration in ApoE^−/−^ model^57^. ADAMTS8 is also expressed in macrophage-rich areas of human atherosclerotic plaques^13^. On the other hand, ADAMTS5 was shown to be markedly reduced in the atherosclerotic aortas in ApoE^−/−^ mice. It may suppress atherogenesis by cleaving versican and biglycan and reducing the lipoprotein retention ability of these proteoglycans^58^. ADAMTS7 has been shown to promote SMC migration which may also have significance in atherogenesis^59^. This work adds ADAMTS4 as another member of the ADAMTS family that contributes to atherosclerosis. Interestingly, there was no obvious compensatory changes of the three closely related ADAMTS4 members – ADAMTS1, -5 and -S8, in both blood circulation as well as atherosclerotic plaques (Supplementary Figs S7 and S8).

In conclusion, removal of ADAMTS4 in ApoE-deficient mice reduces atherosclerosis and enhances plaque stability. This finding is consistent with the reported increase of ADAMTS4 in atherosclerotic plaques and plasma of human CAD patients. Our data demonstrate that ADAMTS4 is not only a biomarker but also a contributor to atherosclerosis. Drugs that target this proteinase may have therapeutic potential in the treatment of atherosclerosis.

## Methods

### Mouse models

ApoE^−/−^Adamts4^−/−^ mice were generated by crossing ApoE^−/−^ mice (Jackson Lab, USA) with Adamts4^−/−^ mice (originated from Jackson lab, USA and supplied by Dr. Satoshi Hirohata, Okayama University), and were both in C57BL/6J background. Genotyping was performed using PCRs as described by Jackson lab. Male and female littermates were fed on a normal chow diet till 6 weeks of age and were then placed on high-fat Western diet (TD.88137; Harlan Teklad, USA) until 12 or 18 weeks of age before being sacrificed by CO_2_ asphyxiation. Animal care, breeding and experimentation was carried out following institutional guidelines approved by the National University of Singapore Institutional Animal Care and Use Committee (NUS IACUC) (Protocol BR11/11 and 075/11) and conform to NIH guidelines.

### Morphometry

Atherosclerosis in the mouse artery (n = 72 arteries) was observed following mouse euthanasia. Briefly the heart was perfused with 50 ml of cold PBS (2X) at physiological pressure and the entire arterial tree was carefully separated from the underlying tissues. Lipid deposition on the aortic arch and carotid arteries was observed in its native state through a stereomicroscope (Zeiss, Germany). Brachiocephalic arteries were excised and processed for immunohistochemical studies soon after whole mount imaging of aorta. Atherosclerotic lesion area was analysed by performing ORO (Sigma, Singapore) *en face* staining of the main trunk of the entire artery devoid of the branches from the aortic root to the iliac bifurcation as described previously with small modifications^20^,^60^. Total plaque area (ORO stained red patches) were calculated using Image J software and total area of arterial trunk were determined. Lesion area (% of total) was obtained by dividing the lesion area to the total arterial trunk area and expressed as percentage of total arterial trunk area.

Thickness of the fibrous cap was measured at its narrowest site. Plaque area was subdivided into a fibrotic cap and a lipid rich necrotic area on the basis of Hematoxylin Eosin staining and confirmed by ORO and Picro-Sirius red staining. Necrotic core was quantified as the unstained area of plaque post DAPI staining. A 3000 μm^2^ area threshold was implemented to avoid counting regions that may not represent substantial areas of necrosis^61^. Cap/Core ratio was calculated as the total area of the fibrous cap to the lipid rich necrotic core^62^.

### Histology

Brachiocephalic arteries were removed and were embedded in optimum cutting temperature compound (OCT) (Tissue-Tek, Netherlands). Complete Brachiocephalic artery (from the base where it touches the arch of aorta to the bifurcation into right common carotid artery) was used for histology studies. Seven or thirty micron thick sections were taken starting from the fork end of the artery to the other end where it touches the main arterial trunk. The sections were placed in 10 different slides (numbered in sequence) with each slide getting the next immediate section. Equidistant slides were used among all the groups for the quantification and comparison. Seven or thirty μm sections were stained with ORO/hematoxylin/Picro-Sirius red staining (ab150681, Abcam, USA). Plaque necrotic core area was measured by DAPI staining of the plaque cross sections and DAPI negative are considered necrotic area.

### Immunohistochemistry

Frozen brachiocephalic artery sections were stained with antibodies against CD68 (marker for macrophages, MCA1957, AbD Serotec, UK), ADAMTS4 (a generous gift from Prof. Hideaki Nagase, Kennedy Institute of Rheumatology, Oxford University, UK), alpha smooth muscle actin (αSMA) (CGA7, Santa Cruz Biotechnology Inc., USA), ADAMTS4/1 cleaved versican V1 neoepitope DPEAEE (ab19345, Abcam, USA) or aggrecan ARGxx neoepitope (ab3773, Abcam, USA) with the corresponding secondary antibody conjugated to horseradish peroxidase (HRP) (Santa Cruz Biotechnology Inc., USA) and visualized by applying 3,3′-diaminobenzidine tetrahydrochloride (DAB) or through Alexa fluor 488 or 568 (Life technologies, Singapore) and visualized under Axiovision compound microscope (Zeiss, Germany) or UltraView Vox Spinning Disk confocal microscopy (PerkinElmer, USA). Percentage positive staining was quantified using Adobe Photoshop CS8 software (USA). The vulnerability index was calculated as the ratio of (ORO stained area + CD68 positive area)/(αSMA area + collagen positive area). The specificity of the antibodies used is demonstrated using isotype antibody controls in all IHC/IF staining (Supplementary Fig. S9).

### Blood sampling and ADAMTS4 immunoassay

The blood samples (0.4 ml) from the C57Bl/6J wild type, ApoE^−/−^ and ApoE^−/−^Adamts4^−/−^ knockout mice were drawn through cardiac puncture into a tube containing 0.1 ml EDTA (0.5 M). The samples were centrifuged at 2,500 rpm for 10 min and the supernatant was carefully transferred and used for ADAMTS4 quantification using an indirect ELISA method. Recombinant human ADAMTS4 (R&D Systems, USA) was used to plot the standard curve. Anti-human ADAMTS4 antibody had a cross reactivity for mouse ADAMTS4 recognizing the identical epitope present in both human and mouse (MAHVDPEEP).

### Cytokine array

The relative expression level of a total of 40 different mouse cytokine proteins in plasma were analysed using proteome profiler array - mouse cytokine array panel A (R&D Systems, USA) as per the manufacturer’s instructions. Relative cytokine expression profile of 18 weeks old ApoE^−/−^Adamts4^−/−^ mice were compared to that of sex- and age-matched ApoE^−/−^ mice. The plasma of 4 mice in each group was pooled and equal amount of total protein (100 μg) was hybridized to the array and compared for relative expression. The experiments were repeated twice (n = 3, each sample contained a plasma pool from 4 different mice). The dot blot density was quantified using Image J software (USA).

### Statistical analysis

Statistical analysis was performed using one-way ANOVA or student’s unpaired t-test (Graphpad PRISM 5, USA). Results are represented as mean ± SEM. Differences were considered as statistically significant if the *P* value is less than 0.05. *p < 0.05; **p < 0.01.

## Additional Information

**How to cite this article**: Kumar, S. *et al*. Loss of ADAMTS4 reduces high fat diet-induced atherosclerosis and enhances plaque stability in ApoE^−/−^ mice. *Sci. Rep.* **6**, 31130; doi: 10.1038/srep31130 (2016).

## Supplementary Material

Supplementary Information

## Figures and Tables

**Table 1:** Plaque characteristics in brachiocephalic artery (n = 5 in all groups, with 5 sections per mouse).

	12 week old mice				18 week old mice			
	Male		Female		Male		Female	
Variable	ApoE^−/−^	ApoE^−/−^Adamts4^−/−^	ApoE^−/−^	ApoE^−/−^Adamts4^−/−^	ApoE^−/−^	ApoE^−/−^Adamts4^−/−^	ApoE^−/−^	ApoE^−/−^Adamts4^−/−^
Plaque Area (0.1× mm^2^)	1.34 ± 0.31	0.91 ± 0.12*	1.00 ± 0.23	0.91 ± 0.11	1.66 ± 0.27	1.08 ± 0.18*	1.58 ± 0.25	1.03 ± 0.20*
Fibrous Cap (μm)	13.25 ± 0.37	15.83 ± 1.41	10.68 ± 0.83	11.88 ± 0.62	10.20 ± 0.39	14.71 ± 1.03*	8.78 ± 0.52	13.15 ± 0.7*
Necrotic Core (0.1× mm^2^)	0.19 ± 0.02	0.06 ± 0.04	0.06 ± 0.01	0.08 ± 0.01	0.46 ± 0.02	0.22 ± 0.02	0.43 ± 0.01	0.24 ± 0.01
Cap/Core Ratio	0.09 ± 0.02	0.14 ± 0.03	0.07 ± 0.01	0.07 ± 0.02	0.05 ± 0.01	0.09 ± 0.01*	0.04 ± 0.01	0.08 ± 0.01*
Lipid content (0.1× mm^2^)	0.66 ± 0.02	0.23 ± 0.04	0.48 ± 0.05	0.42 ± 0.03	0.89 ± 0.09	0.28 ± 0.03	0.78 ± 0.18	0.24 ± 0.02
Lipid content (%)	50.72 ± 2.28	25.81 ± 4.55	48.76 ± 5.57	47.09 ± 4.06	56.15 ± 5.5	25.51 ± 2.55	52.14 ± 11.86	23.95 ± 2.99
CD68+ Area (0.1× mm^2^)	0.56 ± 0.06	0.25 ± 0.02	0.35 ± 0.02	0.33 ± 0.06	0.66 ± 0.08	0.19 ± 0.05	0.63 ± 0.10	0.19 ± 0.03
CD68+ Area (%)	43.64 ± 5.14	28.69 ± 2.05	35.00 ± 1.50	36.94 ± 7.28	41.44 ± 5.16	17.70 ± 5.03	42.08 ± 6.42	19.10 ± 3.11
SMA+ Area (0.1× mm^2^)	0.70 ± 0.05	0.50 ± 0.06	0.52 ± 0.01	0.48 ± 0.01	0.76 ± 0.05	0.70 ± 0.03	0.68 ± 0.06	0.63 ± 0.05
SMA+ Area (%)	54.25 ± 3.45	55.12 ± 6.51	52.72 ± 1.40	54.32 ± 1.92	47.20 ± 3.27	63.94 ± 3.14	45.3 ± 3.76	62.53 ± 5.91
Collagen Content (0.1× mm^2^)	0.75 ± 0.09	0.52 ± 0.05	0.56 ± 0.03	0.52 ± 0.06	0.72 ± 0.08	0.69 ± 0.05	0.50 ± 0.06	0.56 ± 0.14
Collagen Content (%)	57.59 ± 7.13	58.26 ± 6.00	55.55 ± 3.82	58.68 ± 7.19	45.16 ± 5.24	62.82 ± 4.20	33.31 ± 4.06	56.40 ± 14.23
Vulnerability Index	0.86 ± 0.15	0.49 ± 0.07**	0.78 ± 0.06	0.75 ± 0.11	1.06 ± 0.13	0.34 ± 0.05**	1.22 ± 0.31	0.37 ± 0.07**

Data are presented as Mean ± SEM. *p < 0.05, **p < 0.01.
