# Supplementary material for: Loss of ADAMTS4 reduces high fat diet-induced atherosclerosis and enhances plaque stability in ApoE−/− mice
Source: Sci Rep. 2016 Aug 5;6:31130. doi: 10.1038/srep31130 (PMC4974561; doi:10.1038/srep31130)
Supplement: Supplementary Information [file srep31130-s1.pdf]

Supplementary information of

Loss of ADAMTS4 reduces high fat diet-induced atherosclerosis and enhances plaque stability in ApoE$^{-/-}$ mice

Saran Kumar[1#], Mo Chen[1#], Yan Li[1], Fiona H.S. Wong[2], Chung Wee Thiam[2], Md Zakir Hossain[3], Kian Keong Poh[4,5], Satoshi Hirohata[6], Hiroko Ogawa[7], Véronique Angeli[2], and Ruowen Ge[1*].

[1]Department of Biological Sciences, National University of Singapore, Singapore, 117543; [2]Department of Microbiology, Yong Loo Lin School of Medicine, National University of Singapore, Singapore, 117456; [3]Cancer Science Institute of Singapore, Singapore, 117599; [4]Department of Medicine, Yong Loo Lin School of Medicine, National University of Singapore; [5]Department of Cardiology, National University Heart Centre, National University Health System, Singapore 119228; [6]Department of Medical Technology, Graduate School of Health Sciences, Okayama University, Okayama 700-8558, JAPAN; [7]Department of General Medicine, Graduate School of Medicine, Dentistry, and Pharmaceutical sciences, Okayama University, Okayama 700-8558, JAPAN.

*Corresponding author. Tel: +65-65167879; Fax: +65-67792486; Email: dbsgerw@nus.edu.sg

#: These authors contributed equally to this work.

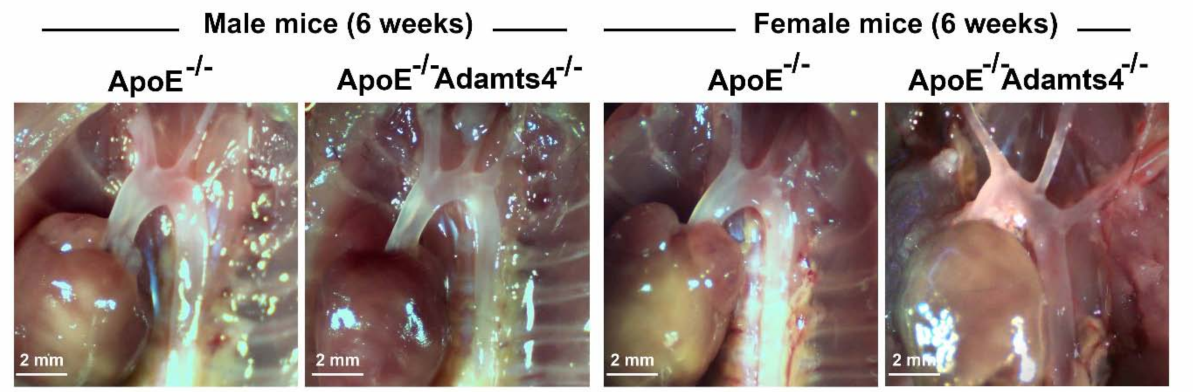

Figure S1. Vessel morphology of 6 weeks old mice. Baseline aortic arch morphology in male and female ApoE$^{-/-}$ and ApoE$^{-/-}$Adams4$^{-/-}$ mice of 6 weeks old before commencement of high fat feeding. No lesion or deformities in vessel morphology was observed.

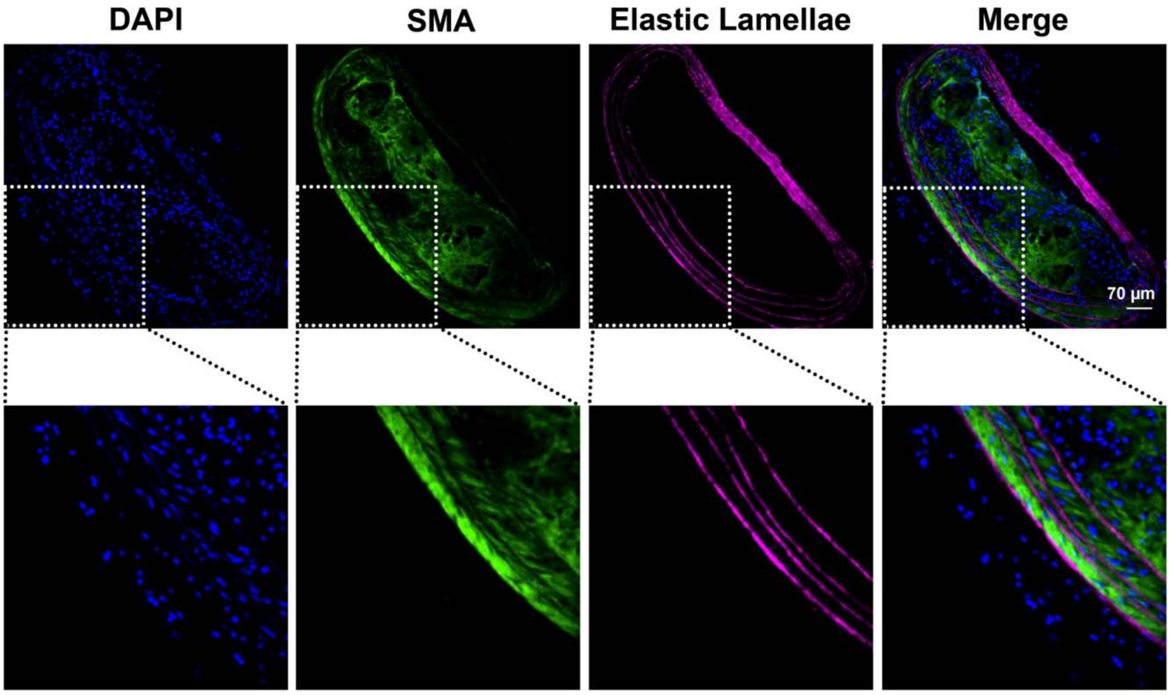

Figure S2. SMA antibody stains smooth muscle cells both in the media of aortic vessel wall and in the plaque. The plaque section was stained by αSMA (CGA7, Santa Cruz Biotechnology Inc., USA) and visualized by corresponding secondary antibody with Alexa fluor 488. The nuclei were counter stained by DAPI. The image was collected by UltraView Vox Spinning Disk confocal microscopy (PerkinElmer, USA). The green channel for SMA and the blue channel for DAPI were scanned under laser power 3% to show the specific staining pattern. The autofluorecent signal from the elastic lamellae was detectable under high laser power without any staining. So we visualized the elastic lamellae in far red channel (empty channel without any staining) under laser power of 50%. The enlarged images in the lower panel clearly show the specificity of the SMA antibody for the smooth muscle cells both in the media and in the plaque. No elastic lamellae were picked up by the SMA staining under the condition to visualize SMA.

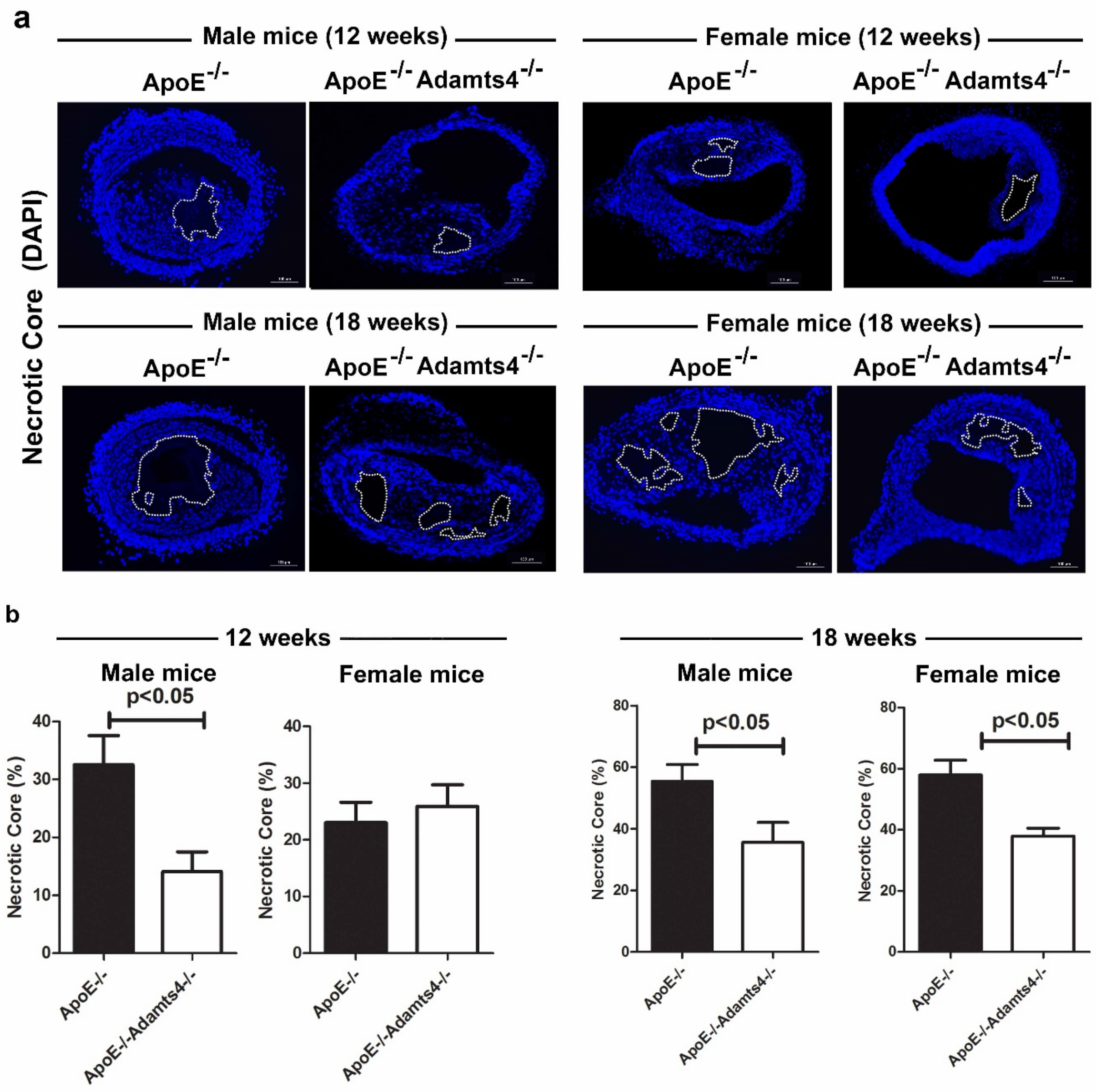

Figure S3. ***Adamts4*** **deficiency reduces the necrotic core area of atherosclerotic lesions at 18 weeks of age in both genders.** (a) Representative fluorescent photomicrographs of DAPI stained cross sections of branchiocephalic artery plaques (necrotic areas outlined with dotted white lines) of ApoE$^{-/-}$ and ApoE$^{-/-}$Adamts4$^{-/-}$ mice at 12 and 18 weeks of age. (b) Quantification of the necrotic core area. n = 5 mice, with 5 sections from each brachiocephalic trunk. Values shown are mean ± SEM.

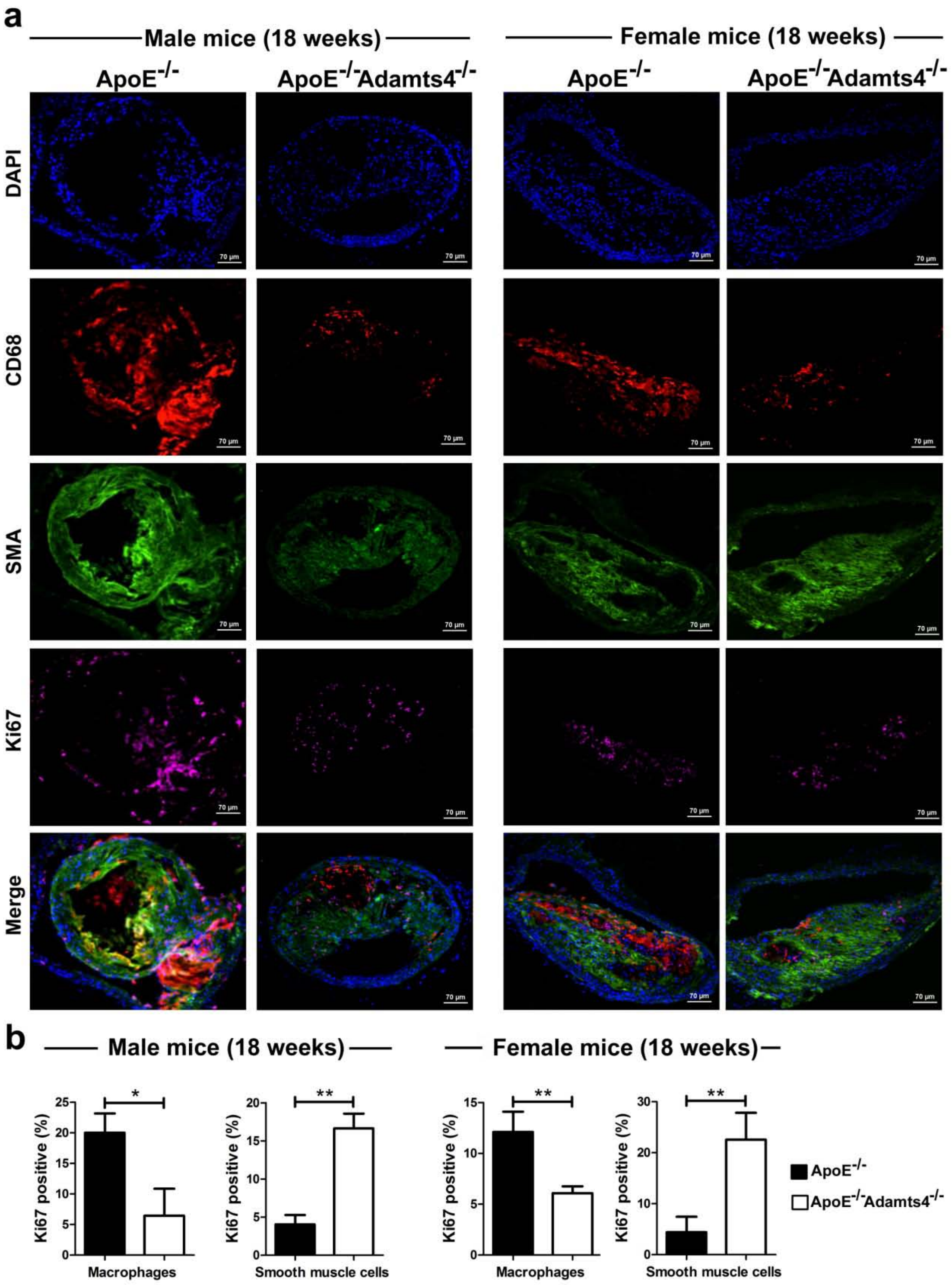

Figure S4. Loss of ADAMTS4 reduces the proliferation of macrophages and induces the proliferation of smooth muscle cells. (a) IF staining of CD68, SMA and Ki67 in the plaques of male and female ApoE$^{-/-}$ and ApoE$^{-/-}$Adams4$^{-/-}$ mice of 18 weeks old post 12 weeks fat feeding. The nuclei were counter stained by DAPI. Ki67 served as the marker for

proliferation. (b) Quantification of the proliferation of macrophages and smooth muscle cells in the plaque area. The proliferation rate of macrophages was calculated by Ki67 and CD68 double positive area against CD68 positive area. The proliferation rate of smooth muscle cells was calculated by Ki67 and SMA double positive area against SMA positive area. n=3 in all groups. Data are presented as Mean ± SD.

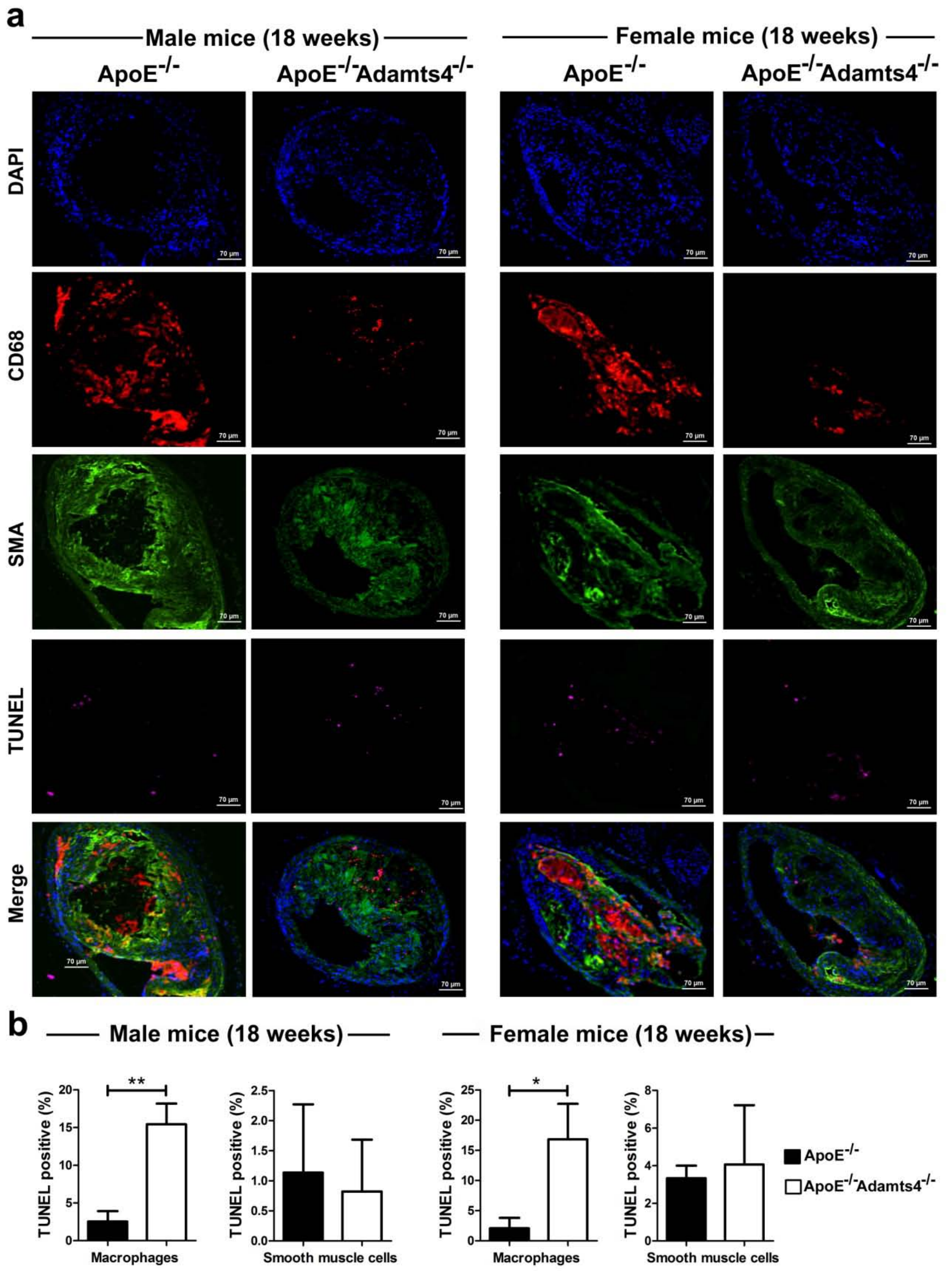

Figure S5. Loss of ADAMTS4 induces the apoptosis of macrophages but has no significant effect on the apoptosis of smooth muscle cells. (a) IF staining of CD68, SMA and terminal deoxynucleotidyl transferase dUTP nick end labelling (TUNEL) in the plaques of male and female ApoE$^{-/-}$ and ApoE$^{-/-}$Adams4$^{-/-}$ mice of 18-weeks old post 12 weeks high

fat diet feeding. The nuclei were counter stained by DAPI. TUNEL signal served as the marker for apoptosis. (b) Quantification of the apoptosis of macrophages and smooth muscle cells in the plaque area. The apoptotic rate of macrophages was calculated by TUNEL and CD68 double positive area against CD68 positive area. The apoptotic rate of smooth muscle cells was calculated by TUNEL and SMA double positive area against SMA positive area. n=3 in all groups. Data are presented as Mean ± SD.

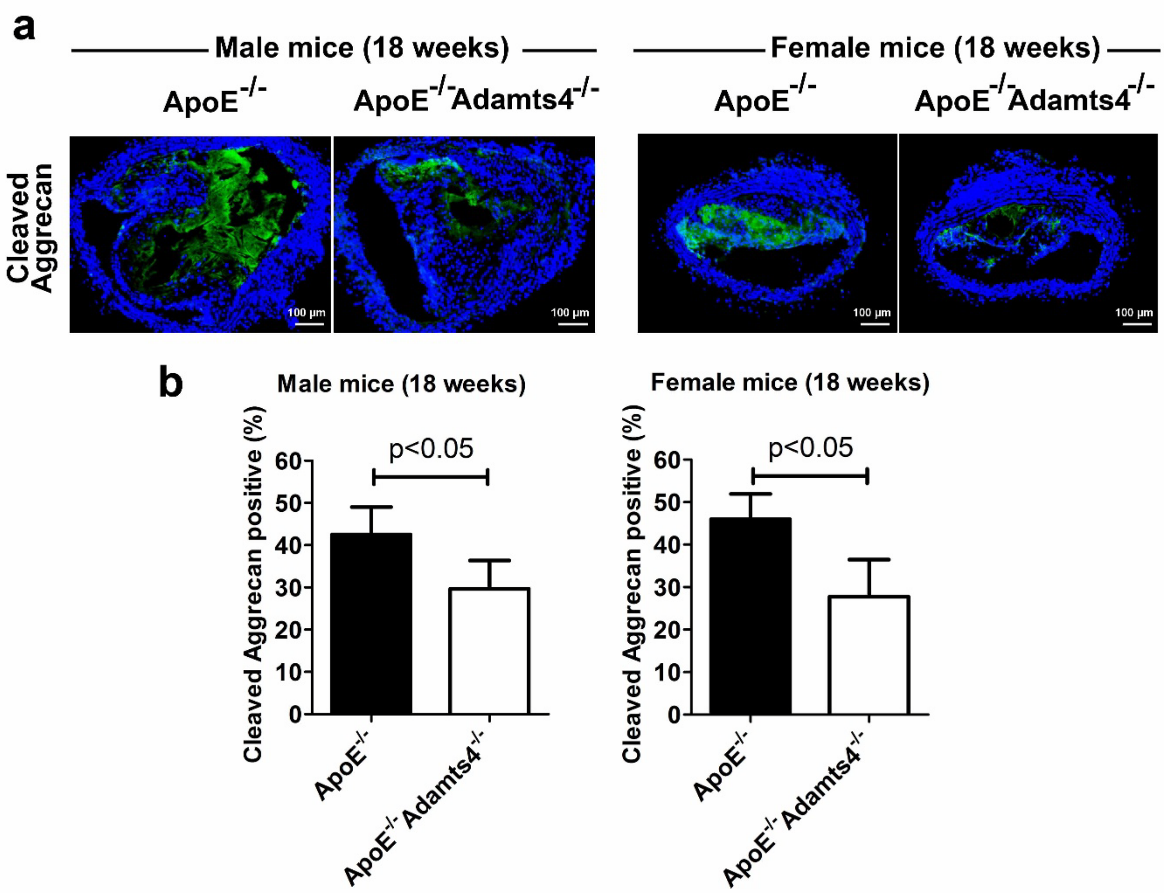

Figure S6. Reduction of aggrecan cleavage in ApoE$^{-/-}$Adams4$^{-/-}$ mice. (a) Representative image of the brachiocephalic trunk section of 18 weeks old mice stained with the neoepitope antibody for cleaved aggrecan. (b) Quantification of cleaved aggrecan in the branchiocephalic plaques. n=5 in all groups. Data are presented as Mean ± SD. Y-axis is the percentage of cleaved aggrecan positive area within the whole plaque area.

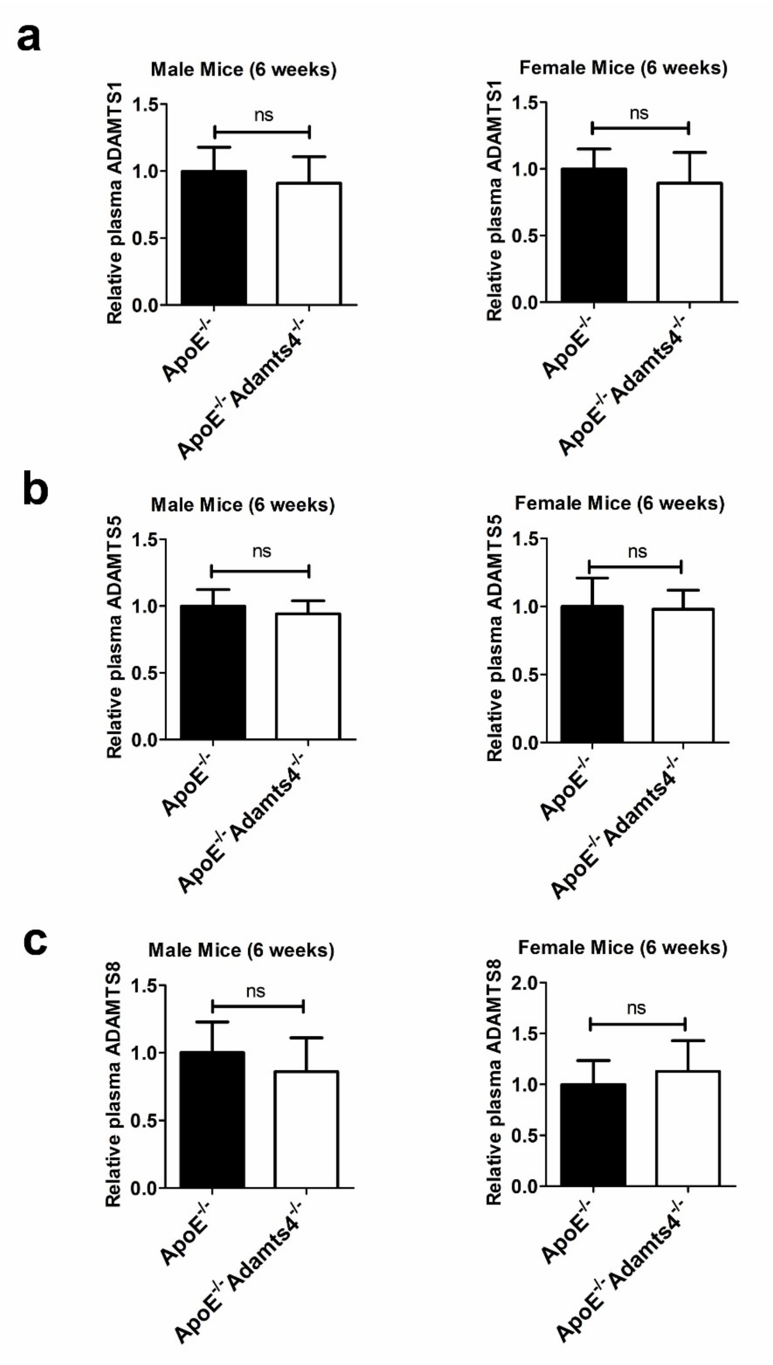

Figure S7. Plasma ADAMTS1, -5 and -8 were not influenced upon ADAMTS4 knockout in 6 weeks old mice before fat feeding. (a) Level of ADAMTS1 in the blood plasma of male and female ApoE$^{-/-}$ and ApoE$^{-/-}$Adams4$^{-/-}$ mice of 6 weeks old determined by ELISA. (b) Level of ADAMTS5 in the blood plasma of male and female ApoE$^{-/-}$ and ApoE$^{-/-}$Adams4$^{-/-}$ mice of 6 weeks old determined by ELISA. (c) Level of ADAMTS8 in the blood plasma of male and female ApoE$^{-/-}$ and ApoE$^{-/-}$Adams4$^{-/-}$ mice of 6 weeks old determined by ELISA. n=3 in all groups. Data are presented as Mean ± SD.

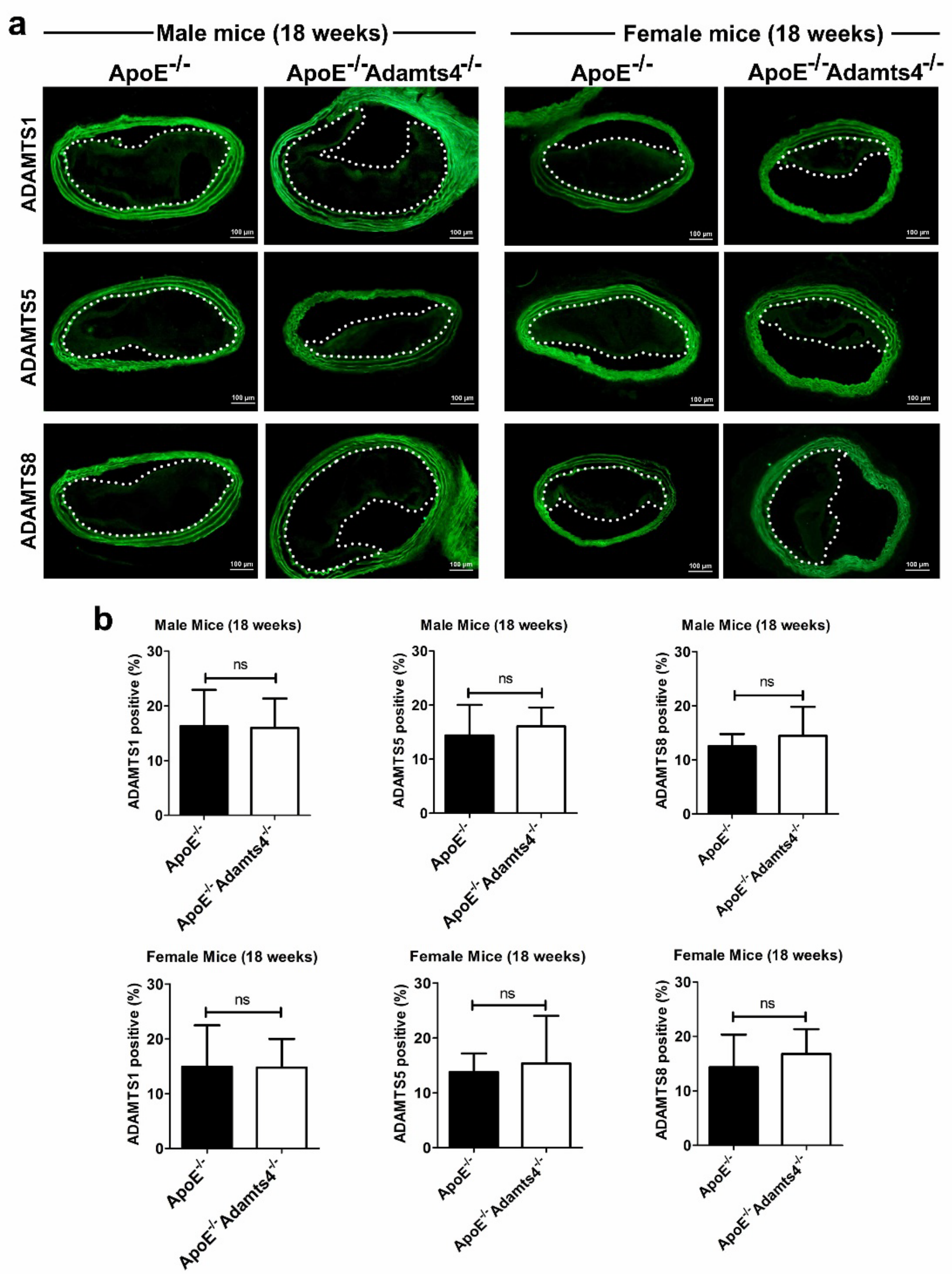

Figure S8. Expressions of ADAMTS1, -5 and -8 in brachiocephalic artery plaques were not altered upon ADAMTS4 knockout in 18 weeks old mice. (a) IF staining of ADAMTS1, ADAMTS5 and ADAMTS8 in the plaques of male and female ApoE$^{-/-}$ and ApoE$^{-/-}$ Adams4$^{-/-}$ mice of 18 weeks old post 12 weeks fat feeding. The plaque area was determined by DAPI staining (not shown) and indicated here by the dashed line. (b) Quantification of the ADAMTS1, ADAMTS5 and ADAMTS8 staining represented as a percentage of stained area over the complete plaque area. n=5 in all groups. Data are presented as Mean ± SD.

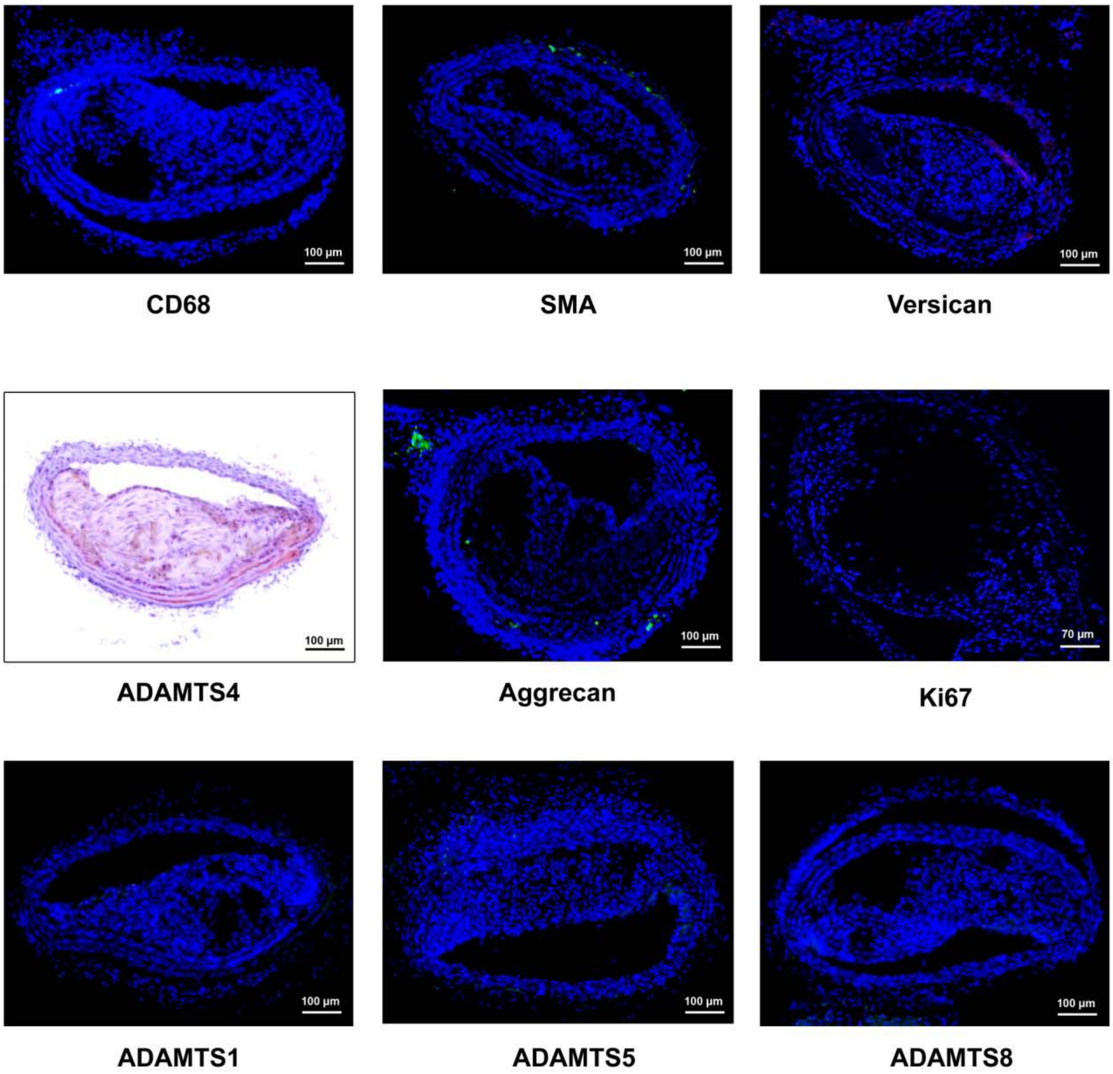

Figure S9. Isotype antibody controls for IHC/IF staining. Sections were stained with corresponding normal isotype specific antibodies (rat-CD68, mouse-SMA and Aggrecan neo-epitope, rabbit-ADAMTS4, ADAMTS1, ADAMTS5, ADAMTS8, Versican neo-epitope and Ki67) followed by secondary antibodies. Nuclei were counter stained by DAPI (for CD68, SMA, ADAMTS1, ADAMTS5, ADAMTS8, Versican, Aggrecan and Ki67) or hematoxylin (for ADAMTS4).

	12 week old mice					18 week old mice				
	Male			Female		Male			Female	
**Mice strain**	**C57BL/6J**	**ApoE$^{-/-}$**	**ApoE$^{-/-}$ Adamts4$^{-/-}$**	**ApoE$^{-/-}$**	**ApoE$^{-/-}$ Adamts4$^{-/-}$**	**C57BL/6J**	**ApoE$^{-/-}$**	**ApoE$^{-/-}$ Adamts4$^{-/-}$**	**ApoE$^{-/-}$**	**ApoE$^{-/-}$ Adamts4$^{-/-}$**
**Body Weight (g)**	30.50 ± 2.80	29.88 ± 2.10	32.94 ± 4.04	23.21 ± 2.14	22.64 ± 2.55	31.31 ± 1.17	30.80 ± 1.6	34.94 ± 2.3	25.97 ± 2.54	25.06 ± 3.24
**Total Cholesterol (mg/dL)**	192.00 ± 37.6	1340.97 ± 285.79	1321.11 ± 195.18	1216.59 ± 112.72	1164.16 ± 120.95	214.06 ± 22.33	1664.62 ± 227.06	1735.93 ± 111.80	1427.34 ± 188.39	1187.16 ± 619.31
**Triglycerides (mg/dL)**	43.61 ± 3.92	110.85 ± 12.15	105.36 ± 7.17	90.73 ± 4.99	91.91 ± 4.71	51.80 ± 2.3	137.04 ± 9.49	139.10 ± 9.03	115.31 ± 12.51	130.31 ± 5.40
**HDL (mg/dL)**	123.55 ± 11.54	42.11 ± 4.56	45.87 ± 4.26	50.73 ± 4.99	45.41 ± 5.32	117.54 ± 6.97	51.80 ± 2.30	59.12 ± 7.43	65.12 ± 21.11	60.31 ± 2.40

Supplementary Table S1. Comparison of body weight, total plasma cholesterol, triglycerides and HDL between ApoE$^{-/-}$Adams4$^{-/-}$ mice and ApoE$^{-/-}$ mice (n=8 in all groups except wild-type where n=5). Data are presented as Mean ± SEM.

Supplementary Methods

Morphometry

Atherosclerosis in the artery of 6 weeks old mice before fat feeding was observed following mouse euthanasia. The heart was perfused with 50 ml of cold PBS (2X) at physiological pressure and the entire arterial tree was carefully separated from the underlying tissues. Lipid deposition on the aortic arch and carotid arteries was observed in its native state through a stereomicroscope (Zeiss, Germany).

Enzyme-linked immunosorbent assay (ELISA)

The blood samples were collected from male and female ApoE$^{-/-}$ and ApoE$^{-/-}$Adamts4$^{-/-}$ mice of 6 weeks old through cardiac puncture of left ventricle after sacrifice (3 mice for each group). Blood plasma (40 µg of total protein) were seeded onto ELISA microplate (Greiner bio-one, Austria) and incubated overnight at 4°C for antigen immobilization. Next day, the plate was blocked using 3% BSA in PBS overnight at 4°C. The following day, the plate was incubated with 50 µl of primary antibody against ADAMTS1 (H-60, Santa Cruz Biotechnology Inc., USA), ADAMTS5 (a generous gift from Prof. Hideaki Nagase, Kennedy Institute of Rheumatology, Oxford University, UK) or ADAMTS8 (H-56, Santa Cruz Biotechnology Inc., USA) (1:500 dilution) for 1 h at room temperature. The wells were washed three times with PBST and the plate was incubated with 50 µl of secondary antibody conjugated with HRP (1:500 dilution) for 1 h at room temperature. After 3 times wash by PBST, the plate was developed by 50 µl of chromogenic substrate TMB (Dako, USA) 5 min and the reaction was stopped by adding 50 µl of 1.25 M sulfuric acid. The absorbance of each well was measured at 450 nm. The level of ADAMTS1, 5 and 8 were plotted by Graphpad PRISM 5 (USA).

Immunohistochemistry

Frozen brachiocephalic artery sections from male and female ApoE$^{-/-}$ and ApoE$^{-/-}$Adamts4$^{-/-}$ mice of 18 weeks old mice post 12 weeks fat feeding were stained with antibodies against ADAMTS1, ADAMTS4, ADAMTS8, aggrecan ARGxx neoepitope (ab3773, Abcam, USA), Ki67 (marker for proliferation, ab15580, Abcam, USA), CD68 (marker for macrophages,

MCA1957, AbD Serotec, UK), and alpha smooth muscle actin (αSMA) (CGA7, Santa Cruz Biotechnology Inc., USA) and corresponding secondary antibodies (Life technologies, Singapore). The apoptotic area in the plaque was determined by Terminal deoxynucleotidyl transferase dUTP nick end labelling (TUNEL) assay using the *in situ* cell death detection kit (Roche, Switzerland) according to the manufacturer's instruction. Nuclei were counter stained by DAPI to determine the plaque area. The images were collected by Axiovision compound microscope (Zeiss, Germany) or UltraView Vox Spinning Disk confocal microscopy (PerkinElmer, USA). Percentage positive staining was quantified using Image J software (USA). The positive staining of ADAMTS1, ADAMTS5, ADAMTS8 or aggrecan ARGxx neoepitope was calculated as the ratio of positive area / plaque area. For the isotype antibody controls for CD68, SMA, ADAMTS4, ADAMTS1, ADAMTS5, ADAMTS8, Versican neo-epitope and Aggrecan neo-epitope antibodies, sections were stained with corresponding normal isotype specific antibodies (rat-CD68, mouse-SMA and Aggrecan neo-epitope, rabbit-ADAMTS4, ADAMTS1, ADAMTS5, ADAMTS8 and Versican neo-epitope) followed by secondary antibodies. Nuclei were counter stained by DAPI (for CD68, SMA, ADAMTS1, ADAMTS5, ADAMTS8, Versican and Aggrecan) or hematoxylin (for ADAMTS4).

Assessment of necrotic core area of atherosclerotic lesions

Necrotic core was quantified as the unstained area of plaque post DAPI staining. A 3000 μm^2 area threshold was implemented to avoid counting regions that may not represent substantial areas of necrosis[1,2]. Cap/Core ratio was calculated as total area of the fibrous cap to the lipid rich necrotic core[3].

Plasma Parameters

Mouse blood was collected through cardiac puncture of left ventricle after sacrifice. The blood plasma were analysed for total cholesterol, HDL and total triglycerides (BioVision Inc., USA).

Statistical analysis

Statistical analysis was performed using one-way ANOVA or student's unpaired t-test (Graphpad PRISM 5, USA). Results are represented as mean ± SEM or mean ± SD as

indicated. Differences were considered as statistically significant if the P value is less than 0.05.

References

1. Seimon, T.A., *et al.* Atherogenic lipids and lipoproteins trigger CD36-TLR2-dependent apoptosis in macrophages undergoing endoplasmic reticulum stress. *Cell metabolism* **12**, 467-482 (2010).
2. Salagianni, M., *et al.* Toll-like receptor 7 protects from atherosclerosis by constraining "inflammatory" macrophage activation. *Circulation* **126**, 952-962 (2012).
3. von der Thusen, J.H., *et al.* Induction of atherosclerotic plaque rupture in apolipoprotein E-/- mice after adenovirus-mediated transfer of p53. *Circulation* **105**, 2064-2070 (2002).
